# Balance equations for physics-informed machine learning

**DOI:** 10.1016/j.heliyon.2024.e38799

**Published:** 2024-10-01

**Authors:** Sandor M. Molnar, Joseph Godfrey, Binyang Song

**Affiliations:** a Institute of Astronomy and Astrophysics, Academia Sinica, Taipei, Taiwan, Republic of China; b Virginia Tech, Falls Church, VA, USA; c Virginia Tech, Blacksburg, VA, USA

**Keywords:** Computational methodologies, Machine learning, Physics-informed systems, Balance equations, Fluid dynamics, Elasticity, Electrodynamics, Thermodynamics

## Abstract

Using traditional machine learning (ML) methods may produce results that are inconsistent with the laws of physics. In contrast, physics-based models of complex physical, biological, or engineering systems incorporate the laws of physics as constraints on ML methods by introducing loss terms, ensuring that the results are consistent with these laws. However, accurately deriving the nonlinear and high order differential equations to enforce various complex physical laws is non-trivial. There is a lack of comprehensive guidance on the formulation of residual loss terms. To address this challenge, this paper proposes a new framework based on the balance equations, which aims to advance the development of PIML across multiple domains by providing a systematic approach to constructing residual loss terms that maintain the physical integrity of PDE solutions. The proposed balance equation method offers a unified treatment of all the fundamental equations of classical physics used in models of mechanical, electrical, and chemical systems and guides the derivation of differential equations for embedding physical laws in ML models. We show that all of these equations can be derived from a single equation known as the generic balance equation, in conjunction with specific constitutive relations that bind the balance equation to a particular domain. We also provide a few simple worked examples how to use our balance equation method in practice for PIML. Our approach suggests that a single framework can be followed to incorporate physics into ML models. This level of generalization may provide the basis for more efficient methods of developing physics-based ML for complex systems.

## 1 Introduction

Machine Learning (ML) models, especially deep neural networks (NNs), offer universal approximation and high expressivity for scientific computing. ML models can approximate solutions to nonlinear Partial Differential Equations (PDEs) by optimizing loss functions without making assumptions about physics, linearization, or local time-stepping [1,2]. While ML models provide compact and expressive representations of complex PDEs, accurately inferring the model parameters is challenging [3] due to high computational costs, high sensitivity to noisy data, and low data efficiencies. However, the solutions obtained for PDEs may violate the initial and boundary constraints as well as the governing equations. Physics-informed ML (PIML) methods have the potential to address these issues by embedding physical laws into the loss functions or the models’ architectures. PML methods allow for the systematic utilization of structured prior knowledge and laws without relying on labeled data from simulations or experiments [[1], [2], [3]]. This approach can be viewed as providing physical guidance and an unsupervised strategy.

There are two fundamental problems of PIML [2]. These problems are formulated in the context of non-linear PDEs. The *first type* is forward inference, a data-driven approach to estimate the solutions of the governing mathematical models. In this case, we seek to *understand* the mathematical models of the PDE [4]. The *second type* is inverse specification, which involves the data-driven identification of the differential operator contained within the PDE. Here, the goal is to *discover* the form of this differential operator, i.e., its terms and parameters [5], from observable data. Following the PIML framework, these two types of problems can be described and formulated using the same equations, but they aim to calculate different variables.

As an illustration of these methods, let us assume, for simplicity, that we have a system dependent only on time described by a function, *u*(*t*). This simplification encompasses a number of interesting cases, such as the Lorenz and Logistic Map systems (e.g., Ref. [6]). In the first type of problems, the physics is known and expressed in the form of a PDE. Recall that to solve a PDE, we need to know the equation and its initial conditions (ICs). Given this information, we create a *loss function*, i.e., a *Lagrangian*, $L_{PDE}\left(u\right)$. The problem we seek to solve is a convex optimization problem: find the minimum of $L_{PDE}\left(u\right)$ as a function of *u*(*t*), assuming that the IC, $u\left(t_{1}\right)=u_{0}$, is known. In ML, NNs are considered universal function approximators for solving such problems [7]. Therefore, the problem can be transformed into a standard optimization problem format.

In the second type of problems, the physics is unknown. We aim to determine the form and parameterization of the differential operator of the PDE. One technique for achieving this is SINDy (sparse identification of nonlinear dynamics; [8]). Similar to the first problem, we seek to solve a convex optimization problem given a loss function, a Lagrangian, while also incorporating constraints to enforce known laws of physics in the problem, e.g., constraints provided by the balance equations.

There is an underlying fundamental principle of physics that enables us to treat basic equations of different branches of classical physics in a unified manner: the principle of balance. This principle states that the change over time of an extensive physical quantity (e.g., number of particles, momentum, or energy) within a fixed volume is due to its net flux through the surface of the volume and its net production within the volume. The equation derived from this principle is called the balance equation. Physics is incorporated into the general framework based on the balance equation through the interpretation of the physical quantities and their fluxes and sources. In this work, the balance equation approach is applied consistently and comprehensively to all areas of classical physics to derive the fundamental equations. The principle of balance provides a unified method for physics-based ML allowing us to model different physical systems using the same algorithm.

The formulation of residual loss terms to enforce the physical laws is at the heart of PIML model construction. While a few papers provide high-level formulations [1,2,5], there is a lack of comprehensive guidance on the formulation of residual loss terms. To bridge this gap, in this paper we propose a new framework that utilizes the balance equation as its foundation to advance PIML development across multiple domains by providing a systematic approach to constructing residual loss terms. The balance equation framework enables the communication of physical constraints to a PINN by specifying the balance equations and the associated constitutive equations. These equations can be combined into a single PDE or a system of PDEs. Each PDE is incorporated into the PINN as a component of the loss function.

Based on our proposed framework, we present an algorithm to solve problems with the PIML method starting from the derivation of the balance equations and the loss terms to the applications of NN. For the inverse problem, we propose to build the loss function based on the balance equations approach. In general, the loss function would contain terms based on the fundamental conservation laws: of mass, momentum, and energy. This approach makes it possible to develop a unified PIML method for inverse problems. We also provide some simple examples of how to use our proposed algorithm for the forward and inverse problems.

The structure of this paper is as follows: In Section 2, we provide a brief overview of PIML methods and display the scope of our proposed new method for incorporating physics. In Section 3, we introduce the concepts of extensive and intensive quantities, as well as the integral and differential forms of the balance equations. The subsequent five sections present applications of the balance equations to different branches of physics. Specifically, we discuss applications in continuous systems, fluid mechanics, elasticity, chemical reactions, and electrodynamics in Sections 4, 5, 6, 7, 8. In Section 9, we demonstrate the efficacy of the balance equation method on simple applications important in physics. In Section 10, we propose an algorithm based on the balance equations that can be incorporated into physics-informed ML methods to analyze complex systems. In this section, as an example, we apply our proposed method to Eulerian hydrodynamics. In Section 11 we provide worked out examples how to implement our method in PIML. Sections 12, 13 contain our summary and conclusions. Additionally, Appendix A. provides a summary of our mathematical notations and Appendix B. summarizes the classifications of second order PDEs.

Throughout this paper, we use the Cartesian coordinate representation for simplicity. We use three-dimensional (3D) vectors and tensors, and therefore their indices are either 1, 2 or, 3. All sums over their indices run from 1 to 3, for example, as *i* in $\sum_{i}=\sum_{i=1}^{3}\;\text{.}$ The ranges for indices in sums involving other quantities are explicitly stated. For example, the equation for energy change under *N* different processes (Equation (9)) contains a sum over *k* = 1, 2, 3, …, *N*, the range of *k* is explicitly stated ($\sum_{k=1}^{N}$).

In order to avoid confusion of notations, the Cartesian coordinates in the *x*, *y*, and *z* directions of a vector, **v**, will be expressed as **v** = (v_x_,v_y_,v_z_) or **v** = (v_1_, v_2_, v_3_). For example, we may use **r** = (*x, y, z*) or **r** = (*x*_1_*, x*_2_*, x*_3_) for the position vector. When it does not cause confusion, we use the “nabla” operator (∇_*i*_ = *∂/∂x*_*i*_) for differential operators to simplify notation. We use only Eulerian control volumes, which are fixed in local coordinates and thus they do not change position in time. To simplify notation, we use script fonts for scalar functions of the position vector, **r**, and time, *t* in order to represent either a scalar field or the *i*th component of a vector valued function (e.g., either S (**r**,*t*) = *s*(**r**,*t*) or S (**r**,*t*) = v_*i*_(**r**,*t*) for *i* = 1, 2 or 3 for a vector field **v**(**r**,*t*)). We assume that the reader is familiar with the basic concepts of the physics we discuss in our paper. Therefore, we provide references only for less well-known results.

## 2 PIML methods and the balance equation approach

In PIML, the governing laws are incorporated into ML models by including PDE residual terms in the loss functions. There are two different approaches to enforce physical initial and boundary constraints. The first approach, known as soft enforcement, penalizes the violation of initial and boundary constraints by integrating penalty terms into loss functions. This approach restricts the space of acceptable solutions [1]. It enables weakly-supervised learning and physically-corrected generation, which require less data to train a model [[9], [10], [11], [12]]. This approach has been widely adopted by physics-informed neural networks (PINNs) [2] and their variants [13,14].

The second approach, known as hard enforcement, imposes initial and boundary constraints by strictly encoding them into the model's architecture. This approach gives rise to another type of PIML models: physics-constrained neural networks (PCNNs) [15]. Within this category, some models impose boundary conditions on neural network weights [[16], [17], [18]], while others incorporate boundary conditions through the padding operations in convolutional neural networks (CNNs) [9,19]. PCNNs enable purely PDE-driven learning that relies solely on physical laws, without the use of labeled data (i.e., data-free training), in a parametric setting [20].

In addition to PINNs and PCNNs, other PIML variants leverage domain decomposition. this approach involves using a separate network for each sub-domain and assigning it to a specific computer node [21,22].

The construction of PIML models integrates physics-based priors into common neural networks to solve PDEs. For example, various physical laws (e.g., enthalpy conservation equation, Navier-Stokes equation, and advection-diffusion equation) have been combined with fully connected neural networks (FCNNs) to solve the associated problems [2,5,15,21,[23], [24], [25], [26]]. Reference [9] incorporated boundary conditions into the model weights of graph neural networks (GNNs) through hard enforcement and included the governing laws in loss functions to solve the Poisson equation, linear elasticity equation, and Navier–Stokes equation. CNNs, combined with enthalpy conservation equations and Poisson equations, have been used to solve various problems such as heat transfer, fluid dynamics, and reactive fluid flows [9,19,27]**.** Researchers have explored the integration of long short-term memory [12] (LSTM) networks with equations of motion through soft enforcement to model nonlinear dynamical systems [14].

Additionally, some generative models have incorporated the enthalpy conservative equation and the Navier-Stokes equation through soft enforcement to generate results from fluid and heat transfer simulations consistent with the laws of physics [12,28]. On this basis, a strand of studies has focused on identifying and overcoming the limitations of basic PIML methods to improve modeling accuracy and convergence. Wang et al. [13] address the failure of current PINN formulations to respect the spatio-temporal causal structure inherent in physical systems by reformulating PINN loss functions to incorporate physical causality explicitly. In another study, the same group of authors [29] address training challenges due to gradient pathologies by proposing a learning rate annealing algorithm that adjusts learning rates based on gradient statistics. Fang [30] proposes a hybrid PIML method that integrates concepts from CNNs and finite volume methods, avoiding automatic differentiation and employing local fitting techniques to approximate the differential operator, thus guaranteeing the convergence rate. Krishnapriyan et al. [31] tackle the challenging optimization landscape caused by PDE-based regularization through curriculum regularization, progressively training the PINN with increasing complexity, and reframing the problem as a sequence-to-sequence task by training the model on smaller time segments sequentially.

In engineering fields, PIML has been widely explored to solve various problems, such as heat transfer [23], fluid dynamics [32,33], power system [34], analyses of internal [35] structures and defects in materials. Many of these applications involve multiple aspects of physics, which can be effectively modeled using PIML. For instance, the combination of heat equation, momentum equation, and continuity equation is utilized to solve multiple-phase Stefan problem [23]. Similarly, advection/convection-diffusion equations, which merge the diffusion and advection/convection equations, describe physical phenomena where particles, energy, or other physical quantities are transferred within a physical system [36,37]. Integrating mass, momentum, and energy equations is essential for addressing flow problems [32,33].

Table 1 provides an overview of PIMLs, including application domains, model architectures, and the governing equations involved. The last column in Table 1 indicates the scope of the proposed balance equation method.

**Table 1:** An overview of PILM including application domains, model architectures, equation types.

Application Domains	Model Architectures	Equation Type	This Paper
Electromagnetism/electrodynamics	FCNN [4,38,39]	Maxwell's eq. Eikonal eq.	✓
Convection, aerodynamic, Quantum mechanics, continuum solid mechanics	FCNN [23]	Continuity eq.	✓
Transient stability, power Systems	FCNN [34]	Swing eq.	
Seismology, electromagnetic radiation, and acoustics	FCNN [30]	Helmholtz eq.	
Macroscopic behavior of many micro-particles in Brownian motion	FCNN [40]	Diffusion eq.	✓
Heat transfer, Stefan problem	FCNN [5,15,23], CNN [9], generative U-Net [12]	Enthalpy conservation	✓
Reaction-diffusion dynamics	FCNN [2]	Allen–Cahn eq.	
Wave propagation	FCNN [5,22,24]	Wave equation, Korteweg–de Vries, Burgers eq. (can be derived from Euler eq.)	✓
Fluid dynamics	FCNN [2,5,15,41], CNN [9], generative networks [28]	Navier-Stokes eq.	✓
Reactive Fluid Flows	FCNN [27,42], CNN [27]	Navier-Stokes + balance equations for elements	✓
Hydraulic dynamics in porous media	FCNN [25,26]	Richards, Darcy's law	✓
Plasma (to find the electric potential)	FCNN [5,21], CNN [9]	Poisson eq.	✓
Viscosity, Diffusion dynamics	Extreme Learning Machine [43], FCNN [21]	Advection/Convection diffusion	✓
Traffic flow, fluid mechanics and gas dynamics	FCNN [41,44]	Burgers (can be derived from Navier-Stokes eq.)	✓
Bubble dynamics	FCNN [45]	Rayleigh-Plesset eq.	
Elasticity	FCNN [46]	Conservation Law	✓
Dynamic structures	LSTM [14]	Equation of Motion	✓
Quantum mechanical systems	FCNN [2]	Shrodinger eq.	
Plasmas, chemical reaction dynamics	FCNN [13]	Kuramoto-Sivashinsky eq.	
Option pricing, hedging	FCNN [47]	Black-Scholes eq.	
Infinite- and finite-horizon optimal control problems	FCNN [48,49]	Hamilton-Jacobi-Bellman eq., Hamilton-Jacobi eq.	
Chaotic Lorenz systems	FCNN [13]	Lorenz eq.	
Migration test	FCNN [40]	Poisson–Nernst–Planck eq.	
Diffusion of charged particles	FCNN [40]	Nernst-Einstein eq.	
Electrode process	FCNN [40]	Butler-Volmer eqs.	
Image processing, computer graphics, computational geometry, optimization, computational fluid dynamics, and computational biology	FCNN [40]	Level set eq.	
Dynamics of the Bose–Einstein condensate	FCNN [50]	Gross–Pitaevskii eq.	

## 3 Premilinaries

### 3.1 Introduction to the balance equation

To illustrate the principle of balance, consider a box containing *N* dice. We may ask: How can the number of dice in the box change? There are two possible ways:

(1) We can add or remove dice from the box, a process that we will refer to as “transporting” dice. The change in the number of dice after this process will be

(1) $$N=N_{in}\;–\;N_{out}\text{.}$$

(2) The number of dice can also change by including a die factory inside the box (imagine a very large box), or having a die recycling center, where the damaged dice are destroyed. The change in the number of dice after these processes would be

(2) $$N=N_{created\;}\;–\;N_{destroyed}\text{.}$$

Considering all of these processes (1 and 2), the change in the number of dice is

(3) $${N=N}_{in}\;–\;N_{out}+N_{created}-N_{destroyed}$$

Some definitions will be helpful. We refer to the net change resulting from adding or removing dice from the box as the *net flux* (or simply *flux*) of dice,

(4) $$F=N_{out}\;–\;N_{in}\text{;}$$

(by convention a positive sign is assigned to the outgoing flux). We refer to the net amount of change due to the creation and destruction of dice as the *net source* (or simply *source*) of dice,

(5) $$S=N_{created}\;–\;N_{destroyed}\text{.}$$

Note that *F* and *S* can both have positive or negative values. With these definitions, the change in the number of dice becomes

(6) N=S–F.

The process we have just illustrated forms the basis of all generic balance equations and, as we will demonstrate, is the foundation of all classical physics equations. If the balance principle is applied globally to an entire system, we use the integral form of the balance equations. If it is applied locally, in a small neighborhood of a point within an extended system, we use the differential form of the balance equations.

The principle of balance may seem too broad, prompting the question: If all the equations of the different branches of classical physics can be derived from a single fundamental equation, then where do the differences between them originate? The answer lies in how we define *N* (i.e., the number of dice), and how we calculate the associated fluxes (*F*) and sources (*S*). For instance, the different definitions for *N*, *F*, and *S* result in the fundamental equations of fluid mechanics, elasticity, and electrodynamics as demonstrated in Sections 5, 6, 8.

While the balance equations are essential, they do not generally form a closed system of equations. They are necessary but not sufficient to specify a system. Additional equations are needed to link the so-called extensive and intensive quantities to form a complete set of equations. These auxiliary equations are commonly referred to as constitutive relations. Constitutive relations describe how extensive and intensive variables depend on the properties of matter. In thermodynamics, an equation of state relates different extensive and intensive state variables. A state variable is an independent variable that describes the state of the thermodynamic system (e.g., pressure, volume, internal energy). The balance equations and the constitutive equations together form a well-defined, closed system of equations that can be solved.

### 3.2 Measurements, extensive and intensive quantities

What does it mean to measure a physical quantity? A physical quantity is measured by a well-defined procedure, enabling comparison with some standard. These standards represent the scale or units of measurement. Although generally in the background, we assume these operational definitions exist and are used to quantify. We then say a measurement determines the scale of a physical quantity once the unit for the measurement is specified, formally: *Q*_*measured*_ = *λ* ∗ [*Q*_*unit*_]. By convention we use square brackets for units of different quantities. For example, we measure the length of a table (e.g., with a ruler) and find it to be 1.5 m, so *λ* = 1.5 in our case, and we write *L*_*table*_ = 1.5∗[L], where the unit length is [L] = 1 m. Note, we could use 2 m, 10 cm, etc., as a unit length for measurement. Once we choose the unit length, all the quantities derived from length, have their units fixed; for example, the unit of volume will be [V] = [L^3^].

In everyday life, we distinguish between quantities, for example 1 apple, 3 apples, etc, and qualities, red apples, green apples, etc. In physics, we distinguish between two fundamentally different types of quantities: *extensive* and *intensive quantities*, which are analogous to the usual quantities and qualities in everyday speech.

In general, consider a physical system in equilibrium, *S*({*y*_*i*_}; {*X*_*i*_}), which depends on a set of intensive, {*y*_*i*_}, and extensive, {*X*_*i*_}, quantities, where the curly brackets indicate a set of variables with *i* = 1,2*,…,N*. Let us divide *S* into two partitions, *S*_*A*_({*y*_*i*_^*A*^}; {*X*_*i*_^*A*^}) and *S*_*B*_({*y*_*i*_^*B*^}; {*X*_*i*_^*B*^}). Quantities that are additive, *X*_*i*_ *= X*_*i*_^*A*^ + *X*_*i*_^*B*^, are called extensive quantities. In other words, extensive quantities can be scaled with the size of the system. We call intensive quantities those which do not depend on the size of the system, i.e., *y*_*i*_ ≠ *y*_*i*_^*A*^ + *y*_*i*_^*B*^. For example, the temperature, *T*, is an intensive quantity since it is not equal to the sum of the temperatures of the two subsystems, *T ≠ T*_1_ + *T*_2_, but *T* = *T*_1_ = *T*_2_ (since we assumed that the system is in equilibrium). Moreover, intensive quantities can be assigned to a point within the system. For example, temperature can be defined at any points within the system in equilibrium.

A derived intensive quantity, *y*, is a homogeneous function of degree zero of its extensive variables, i.e., for all *λ*,

(7) $$y=F\left(\left\{y_{i}\right\},\left\{\lambda X_{j}\right\}\right)=\lambda^{0}F\left(\left\{y_{i}\right\},\left\{X_{j}\right\}\right)=F\left(\left\{y_{i}\right\},\left\{X_{j}\right\}\right)\text{.}$$

A derived extensive quantity, *X*, is a homogeneous function of degree one of its extensive variables, i.e., for all *λ*,

(8) $$X=G\left(\left\{y_{i}\right\},\left\{\lambda X_{j}\right\}\right)=\lambda^{1}G\left(\left\{y_{i}\right\},\left\{X_{j}\right\}\right)=\lambda \;G\left(\left\{y_{i}\right\},\left\{X_{j}\right\}\right)\text{.}$$

We can obtain derived intensive quantities by dividing one extensive quantity by another. In general, every extensive quantity, *X*, has an associated density: *ρ*_*X*_ = *X/V*, where *V* is the volume of the system. For example, mass density is a combined intensive quantity obtained by dividing mass by volume, *ρ* = (*λm*)*/*(*λV*) = *λ*^0^(*m/V*). On the other hand, multiplying an extensive quantity by an intensive quantity result in an extensive quantity. For example, multiplying mass by velocity yields an extensive combined quantity, the momentum, **p** = (*λm*)**v** = *λ*(*m***v**). Note that velocity must be an intensive variable since scaling a system by *λ* does not changes its velocity, and it can be assigned to a point within a system.

### 3.3 Physical meaning of extensive and intensive quantities

The distinction between intensive and extensive quantities provides insight into the energetic structure of the system. Experiments indicate that a small change in the energy of a system in equilibrium is proportional to the change in the corresponding extensive quantity (e.g., Ref. [51]). This leads us to the following definition: we define the *basic intensive quantity*, *y*, as a coefficient, which determines the amount of energy change, Δ*E*, resulting from small change in the corresponding *basic extensive quantity*, *X*. In the case of *N* different processes (*k* = 1, 2, 3, …, *N*), a small energy change is the sum of all terms,

(9) $$\Delta E=\sum_{k=1}^{N}\;y_{k}\;\Delta X_{k}\text{.}$$

We say that *y*_*k*_ and *X*_*k*_ are *energy conjugate* (as opposed to, e.g., entropy conjugate) intensive and extensive variables of process *k*, since their product has the dimension of energy. In other words, they are related to each other by a Legendre transformation. The basic extensive variables, *X*_*k*_, are also called *natural variables* in thermodynamics (e.g., entropy, volume, number of particles). A list of conjugate extensive and intensive quantities for different processes is given in Table 2.

**Table 2:** Conjugate extensive and intensive quantities for different processes.

Process	Intensive quantity	Extensive quantity	Change in energy
Thermal	Temperature	Entropy (S)	T ΔS
Mechanical	Negative pressure (-*p*)	Volume (V)	-*p*ΔV
	Stress tensor (*σ*_ij_)	Volume strain tensor (V*ε*_ij_)	$\sum_{ij}\sigma_{ij}\left(V\Delta \varepsilon_{ij}\right)$
	Surface tension (*σ*_*S*_)	Area (A)	*σ*_*S*_ΔA
	Linear tension (*τ*_*L*_)	Length (L)	*τ*_*L*_ΔL
Chemical	Chemical potential (*μ*_i_)	Number of species *i* (*N*_*i*_)	*μ*_*i*_Δ*N*_*i*_
Gravitational	Gravitational potential (*Φ*)	Mass (m)	*Φ*Δ*m*
Electrostatic	Electrostatic potential (*U*_q_)	Electric charge (*q*_*e*_)	*U*_q_ Δ*q*_*e*_
Electric polarization	Electric field strength (**E**)	Total electric polarization (**P**)	**E** · **ΔP**
Magnetic polarization	Magnetic field strength (**H**)	Total magnetization (**M**)	**H** · **ΔM**

The energy of a system is a derived extensive quantity because it is a homogeneous function of degree one of its extensive variables. As a consequence of Equation (9), we obtain the energy conjugate intensive quantity, $y_{k}$, associated with the extensive quantity, $X_{k}$, as a derivative of the energy function, *E*(*X*_1_*,…,X*_*k*_*, …,X*_*N*_), with respect to *X*_*k*_,

(10) $$y_{k}=\frac{\partial E}{\partial X_{k}}\text{.}$$

Every process is associated with relevant intensive quantities. If any of the relevant intensive quantities, *y*_*k*_, does not have a uniform value throughout a closed continuous system (e.g., if the temperature varies with position), the system is not in equilibrium. Changes will occur due to *generalized forces*, generated by the inhomogeneities of *y*_*k*_, causing *generalized displacements*, Δ*X*_*k*_, in its associated extensive variables. For example, temperature inhomogeneities cause changes in entropy (the energy conjugate of temperature) and energy in a system. Note that all relevant processes must be taken into consideration.

The direction of change induced by inhomogeneities in the relevant intensive variables will drive the system toward equilibrium. Consequently, the intensive quantities will undergo changes, but the sum of the extensive quantities in a closed system (e.g., the total mass and volume of the system) will remain constant.

### 3.4 Duality between extensive and intensive variables

The forward and inverse problems in PIML can be regarded as dual, in a way that will be described now.

The abstract definition of tangent and cotangent spaces is informative for the distinction between Lagrangian and Hamiltonian representations, and the corresponding distinction between intensive and extensive variables. From the point of view of physics, the Lagrangian representation uses the tangent space, while the Hamiltonian representation uses the corresponding cotangent space. The Lagrangian describes a kinematic relationship among intensive variables while the Hamiltonian describes a dynamical relationship between extensive variables. This distinction becomes clear when one considers the formal relationship between the Lagrangian $L\left(q,\dot{q},t\right)$ and the Hamiltonian H(p,q,t), i.e., the Legendre transformation:

(11) $$H\left(p,q,t\right)=\sum_{i}p_{i}{\dot{q}}^{i}-L\left(\dot{q},q,t\right)\text{.}$$

The Lagrangian is expressed entirely in terms of kinematic variables (configuration space), while the Hamiltonian is expressed in terms of dynamical variables (phase space). The Hamiltonian is extensive in the sense that momentum, p, is additive, while the Lagrangian is intensive in the sense that velocity, $\dot{q,}$ is not (generally) additive. That velocity is not generally additive is particularly clear when one considers the case of relativity, where momentums add but velocities do not (see Section 3.2).

We apply these concepts to differential systems. Extensive variables depend on the size or quantity of the system, e.g., in a thermodynamic system we have the volume, the internal energy, and the number of particles. They scale with the size of the system. Intensive variables do not depend on the size of the system (e.g., temperature, pressure, and chemical potential; see Section 4.1); they remain the same regardless of the size of the system.

At a given point in the state space, the *tangent space* represents all possible changes (differentials) of the extensive variables. For example, dV,dU, and dN are elements of the tangent space. At the same given point in the state space, the *cotangent space* represents all linear functionals of the extensive variables, which are typically functions of the intensive variables. These differentials can be seen as linear functionals acting on the tangent vectors. For example, dP, dT, and dμ are elements of the cotangent space.

These two spaces are related by pairing differentials of the intensive variables with linear functions of the differentials of extensive variable, e.g., the differential form of internal energy dU=TdS−PdV+μdN, the intensive variables T,−P, and *μ* act as coefficients (linear functionals) of the differentials of the extensive variables dS,dV, and dN.

Geometrically, if we imagine the extensive variables forming a manifold, i.e., the space of integral curves of a system, then the tangent space at any point on this manifold represents all possible infinitesimal changes in the extensive variables. The cotangent space, being the dual, consists of linear functionals that measure these changes. Intensive variables, in this context, can be viewed as elements of the cotangent space that evaluate how these extensive variables change.

### 3.5 Flux densities and flow rates

In transport phenomena, *fluxes* represent the *flow rates* of extensive quantities, *X*, across a fixed surface area with dimensions of [units of X]/[time] or [X] [T]^−1^. *Flux densities* are defined as flow rates of extensive quantities over a surface area with dimensions of [units of extensive quantity]/([time] [area]) or [X] [T]^−1^ [L]^−2^. Often, the terms *fluxes* and *flux densities* are used interchangeably in transport theory. Thus, to avoid confusion, in the rest of our paper, we use the term “flow rates” for quantities with dimensions of [X] [T]^−1^, and “fluxes” in general to describe the specific process quantities being transported.

#### 3.5.1 Flux densities of extensive quantities

Fluxes of extensive quantities can arise from being carried by a flow (e.g., particles suspended in a fluid), known as *convective transport*, or from differences in their corresponding intensive quantities, known as *conductive transport*.

The equations for the transport of a scalar extensive quantity and the *i*th component of a vector extensive quantity are similar. To simplify some equations, we use script fonts, X, to refer to either a scalar, *X*, or an *i*th component, *X*_*i*_, of a vector extensive quantity, **X**. We use J to represent a vector quantity representing either the flux density, J = **j**, of a scalar extensive quantity, *X*, or the *i*th raw, J = **J**_**i**_, of a second-rank tensor flux density, **J**, of a vector extensive quantity, **X**. **J**_**i**_ describes the transport of the *i*th component of **X** in the *x*, *y*, and *z* directions,

(12) $$J_{i}\left(r,t\right)=\left[J_{i1},J_{i2},J_{i3}\right]\text{.}$$

The flux density is a sum of the convective and conductive flux densities, which, using our notation, can be expressed as

(13) J(r,t)=J(rcv,t)+J(rcd,t),

or for tensor flux densities, **J**, we may use the vectorial notation,

(14) $$J\left(r,t\right)=J_{\text{cv}}\left(r,t\right)+J_{\text{cd}}\left(r,t\right)\text{.}$$

The *convective flux density* of an extensive quantity is the flow rate of *X* (or *X*_*i*_), over a fixed surface, *A*, with dimensions of [X][T]^−1^[L]^−2^. The convective flux density of X is a vector parallel to the flow velocity, **v** = [v_1_,v_2_,v_3_], carrying *X (*or *X*_*i*_),

(15) J(rcv,t)=ρX(r,t)v(r,t),

where $\rho_{X}$ is either $\rho_{X}$ = *ρ*_*X*_, the density of a scalar extensive quantity, *X*, or $\rho_{X}$ = (*ρ*_*X*_)_*i*_, the density of the *i*th component, *X*_*i*_, of a vector extensive quantity, **X**. Note that calculating the flux density using Equation (15) gives the correct dimension: [X]/[T]/[L]^2^.

The convective flux density of a scalar extensive quantity, **j**_cv_(**r**,*t*), with density *ρ*_*X*_(**r**,*t*), is a vector quantity,

(16) $$j_{\text{cv}}\left(r,t\right)=\rho_{X}\left(r,t\right)\;v\left(r,t\right)\text{.}$$

The convective flux density of a vector extensive quantity, **J**_cv_(**r**,*t*), with density *ρ*_**X**_(**r**,*t*), is a second-rank tensor,

(17) $$J_{\text{cv}}\left(r,t\right)=\rho_{X}\left(r,t\right)⊗v\left(r,t\right)\text{,}$$

in component notation, the *i*th component is

(18) $${\left(J_{cv}\right)}_{ij}={\left(\rho_{X}\right)}_{i}v_{j}\text{,}$$

where we used the definition of the outer product of two vectors, (**a** ⊗ **b**)_*ij*_ = *a*_*i*_*b*_*j*_. The *i*th row of the convective flux density, Equation (17),

(19) $$J_{\mathrm{cvi}}={\left(\rho_{X}\right)}_{i}\;v=\left[\rho_{Xi}\;v_{1},\rho_{Xi}\;v_{2},\rho_{Xi}\;v_{3}\right]\text{,}$$

describes the flow of the *i*th component of ***ρ***_**X**_ in the *x*, *y*, and *z* directions.

In the case of a small change in the energy of a system in equilibrium, the *conductive flux density*, **j**_**cd**_(**r**,*t*), of an extensive quantity, *X*, generated by its *conjugate* intensive quantity, *y*(**r**,*t*), may be considered to be proportional to a small difference in *y*, *j*_*cd*_ = *L*Δ*y*, where *L* is the conduction coefficient associated with *y* (e.g., Ref. [51]).

The inhomogeneities of a scalar intensive quantity, *y*(**r**,*t*), at a fixed time, can be measured by its spatial derivatives. Assuming small variations in *y*, the change in *y* may be approximated by its first derivative, (*∂*_1_*y,∂*_2_*y,∂*_3_*y*). In the so-called *linear approximation*, we also assume that the conduction coefficients, *L*, does not depend on the associated intensive quantity, *y*. In this approximation, the *i*th component of the conductive flux density for a scalar intensive quantity, *y*(**r**,*t*), can be expressed as

(20) $${\left(j_{cd}\right)}_{i}\left(r,t\right)=\sum_{j}L_{ij}\left(r,t\right)\partial_{j}y\left(r,t\right)\text{,}$$

where *L*_*ij*_ -s are the components of a 2nd-rank tensor called the *transport* or *conduction coefficients* for *X*. Using the definition of multiplication of a 2nd-rank tensor, **T**, with a vector, **w** (Equation (A1)), we can write Equation (20) in vector notation as

(21) $$j_{\text{cd}}\left(r,t\right)=L\left(r,t\right)\;\text{grad}\;y\left(r,t\right)\text{,}$$

where the *i*th row of **L**, **L**_**i**_ = [*L*_*i*1_*,L*_*i*2_*,L*_*i*3_], describes the conduction of *X* due to the inhomogeneities of *y*(**r**,*t*) in the (*x, y, z*) directions.

The conduction coefficients depend on the properties of the material. In the context of the balance equation method, the fluxes are provided by the constitutive relations, which link the material variables to the fluxes. Thus, Equation (20) for the conduction coefficients is also called a constitutive equation. For example, the constitutive relation for for the mass flux density due to diffusion (Equation (161)) and Equation (169) for the flux density due to heat conduction.

We refer to a material as a *linear material*, if its conduction coefficients do not depend on its associated intensive quantities and the generated fluxes are linear in the derivatives of their associated intensive quantities. In *isotropic materials*, the conduction coefficients do not depend on direction. For such materials, a scalar conduction coefficient, *L*_*X*_, can be introduced to describe the conduction for a scalar extensive quantity, *X*. In *linear isotropic materials L*_*X*_ = *L*_*X*_(**r**,*t*) does not depend on *y*, thus the flux density associated with *X* can be written as

(22) $$j_{\text{cd}}\left(r,t\right)=L_{X}\left(r,t\right)\;\text{grad}\;y\left(r,t\right)\text{.}$$

The assumption that a material is linear and isotropic is a frequently used approximation in physics and engineering.

The flux density of a scalar extensive quantity is the sum of the convective and conductive flux densities. In the linear isotropic approximation, the sum becomes (Equations (15), (22)),

(23) $$j\left(r,t\right)=\rho_{X}\left(r,t\right)\;v\left(r,t\right)+L_{X}\left(r,t\right)\;\text{grad}\;y\left(r,t\right)\text{.}$$

Similarly to the case of a scalar extensive quantity, the conductive flux density tensor, **J**_cd_(**r**,*t*), of a vector extensive quantity, **X**, is generated by the inhomogeneities of its conjugate vector intensive quantity, **y**(**r**,*t*). Small spatial variations in a vector field, such as **y**(**r**,*t*), can be measured by its partial derivatives. In the linear approximation, the conductive flux density tensor, **J**_cd_, is proportional to the partial derivatives of **y** and the conduction coefficients do not depend on **y**. Thus, the flux density can be expressed as

(24) $${\left(J_{cd}\right)}_{ij}\left(r,t\right)=\sum_{mn}L_{ijmn}\left(r,t\right)\;\frac{\partial y_{m}}{\partial x_{n}}\text{,}$$

where *L*_*ijmn*_ are components of a 4th-rank conduction tensor (note the implicit summation over *m* and *n* = 1, 2, 3), and *∂y*_*m*_*/∂x*_*n*_ is the tensor gradient operator, Grad, acting on a three-dimensional (3D) vector field (Equation (A7)). The transport of *X*_*i*_ by conduction in the *x*, *y*, and *z* directions is described by the *i*th row of **J**_cd_,

(25) $$J_{\text{cd}i\;}=\left[J_{i1},J_{i2},J_{i3}\right].$$

Note that instead of the letter **J**, **T** is also used to refer to tensor flux densities. We may refer to *L*_*ijmn*_ as generalized conduction coefficients, since Equation (24) is a generalization of Equation (20) to vector intensive quantities.

In general, *L*_*ijmn*_ has 81 (3^4^) independent components in 3D. In isotropic materials, **L** is an isotropic tensor, as rotating the coordinate system should not alter **J**_cd_. Requiring that *L*_*ijmn*_ to be invariant under rotation reduces the 81 independent components to three. Further assuming that the resulting tensor, **J**_cd_, should be symmetric, reduces the number of independent coefficients of **L** to two. Consequently, we may decompose the conductive flux density tensor, **J**_cd_, as:

(26) $$J_{\text{cd}\;}=L_{1\;}\varepsilon \left(y\right)+L_{2\;}\left(\mathrm{div}\;y\right)I_{3},$$

where (*I*_3_)_*ij*_ = *δ*_*ij*_ is the 3D unit matrix (*δ*_*ij*_ is the *ij*-th component of the Kronecker **δ** function), *L*_1_ and *L*_2_ are conduction coefficients (e.g., related to the Lamé parameters *λ* and *μ*: *L*_*1*_ = –2*μ* and *L*_*2*_ = –*λ*; Equation (64)), and **ε**(**y**) is the *generalized strain tensor* (e.g., strain tensor of elasticity, ε(u); Equation (105)),

(27) $$\varepsilon \left(y\right)=\frac{1}{2}\left[\text{Grad}\;y+{\left(\text{Grad}\;y\right)}^{T}\right],$$

where superscript T denotes the transpose of the differential operator, Grad **y** (Equation (A7)), in component notation the (*i, j*)th component is expressed as

(28) $$\varepsilon_{ij}=\frac{1}{2}\left[\frac{\partial y_{i}}{\partial x_{j}}+\frac{\partial y_{j}}{\partial x_{i}}\right].$$

Note that there is no summation over *i* and *j*. The tensors **ε** and **I**_3_ satisfy the aforementioned symmetry requirements. It can be shown that **ε**(**y**) cannot contain other terms. Another approach to obtain this result is to recognize that there are only two 2nd-rank tensors containing the first derivatives of **y**, which satisfy the conditions of isotropy and symmetry, these are **ε**(**y**) and (div **y**)**I**_3_.

In isotropic materials, in the linear approximation, we obtain the total flux density of **X** by adding the convective and conductive transport, Equations (17), (26),

(29) $$J=\rho_{X\;}⊗v+L_{1\;}\varepsilon \left(y\right)+L_{2\;}\left(\mathrm{div}\;y\right)I_{3},$$

in component notation,

(30) $$J_{ij}={\left(\rho_{X}\right)}_{i}v_{i}+L_{1}\varepsilon_{ij}\left(y\right)+L_{2}{\;\delta }_{ij}\sum_{k}\partial_{k}y_{k}\text{.}$$

Note that *J*_*ij*_ is a scalar function; it represents one component with row and column indices of *i* and *j* (*i, j* = 1, 2, or 3) of the Cartesian coordinate representation a second-rank tensor, **J**,. Whereas **J**_**i**_ is a vector, specifically the *i*th row of the coordinate representation of **J**. We summarize the flux densities of scalar and vector extensive quantities in isotropic materials in Table 3.

**Table 3:** Flux densities and flow rates (fluxes) of scalar and vector extensive quantities.

Extensive	Associated intensive	Flux densities (convective + conductive)		Flow rates (fluxes)
quantity	quantity	Convective	Conductive (linear, isotropic)	
Scalar (X)	Scalar (*y*)	**j**_cv_ = *ρ*_*X*_**v**^a^	**J**_cd_ = *L*^b^ grad **y**	*F*(*t*) = $\int_{A}\;j\cdot \mathrm{dA}$
		(*j*_*c*v_)_*i*_ = *ρ*_*X*_ v_*i*_	(*j*_*cd*_)_*i*_ = *L ∂*_*i*_*y*	*F*(*t*) = $\sum_{i}\;\int_{A}j_{i}{\left(dA\right)}_{i}$
Vector (**X**)	Vector (**y**)	**J**_cv_ = ***ρ***_**X**_ ⊗ **v**^c^	**J**_cd_ = *L*_*1*_**ε**(**y**) + *L*_*2*_ (div **y**) **I**_**3**_^d^	**F**(**t**) = $\int_{A}\;J\mathrm{dA}$
		(*J*_*c*v_)_*ij*_ = (*ρ*_*X*_)_*i*_ v_*j*_	(*J*_*cd*_)_*ij*_ = *L*_*1*_*ε*_*ij*_(**y**) + *L*_*2*_*δ*_*ij*_$\sum_{k}$*∂*_*k*_*y*_*k*_	*F*_*i*_(*t*) = $\sum_{j}\;\int_{A}j_{ij}{\left(dA\right)}_{j}$

a *ρ*_*X*_ and **v** are the density associated with the scalar extensive quantity, *X*, and the velocity of the fluid carrying *X*.

b *L* is the conduction coefficient of *X* in isotropic materials.

c *ρ*_**X**_ and **v** are the density (a vector) associated with the vector extensive quantity, **X**, and the velocity of the fluid carrying **X**.

d **ε**(**y**) is the strain tensor, *ε*_*ij*_ = (1*/*2)(*∂*_*i*_*y*_*j*_ + *∂*_*j*_*y*_*i*_) (Equation (28)); *L*_1_ and *L*_2_ are the conduction coefficients of **X** in isotropic materials.

The conductive flux densities are proportional to the inhomogeneities expressed as derivatives of the associated intensive quantities. In the linear approximation, these conductive flux densities are directly proportional to the first derivatives of the intensive quantities and the conduction coefficients are independent of the associated intensive quantities. In linear isotropic materials, the conduction coefficients for scalar intensive quantities are scalar values (Equation (22)), while those for vector intensive quantities are characterized by two independent coefficients (Equation (26)).

#### 3.5.2 Flow rates (fluxes) of extensive quantities

We use script fonts to represent the total flow rate, or flux, F = *F*, or F = *F*_*i*_, of either the flux rate of a scalar extensive quantity, *X*, or the flux rate of the *i*th component of a vector extensive quantity, **X,** across a surface area *A*. The flux rate can be calculated as a surface integral of the flux densities over *A*,

(31) $$F\left(t\right)=\int_{A}J\left(r,t\right)\cdot \mathrm{dA,}$$

where **dA** is the normal vector to the differential surface element pointing outward if the surface is closed (by convention), and J (**r**,*t*) = **j**(**r**,*t*) or J (**r**,*t*) = **J**_**i**_(**r**,*t*) represents the flux density vector of a scalar or the *i*th component of a vector extensive quantity (Equation (12)) at position **r** and time *t*. Note that the surface, *A*, needs to be orientable for the integral to be meaningful. Note that J (**r**,*t*) must be only continuous (except on a set of measure zero on the surface *A*), not necessarily differentiable, for the integral to exist.

The flow rate (flux) for vector extensive quantities can be written in vector notation as

(32) $$F\left(t\right)=\int_{A}J\left(r,t\right)\;\mathrm{dA.}$$

In component notation, the *i*th component becomes

(33) $$F_{i}\left(t\right)=\sum_{j}\int_{A}J_{ij}{\left(dA\right)}_{j}\text{,}$$

where *i* = 1, 2, 3, corresponding to the three spatial dimensions and we sum over *j* = 1, 2, 3. The multiplication of a 2nd-rank tensor (**J**) and a vector (**dA**) is defined in Equation (A1). The 5th column in Table 3 shows the flow rates (fluxes) of scalar and vector extensive quantities.

### 3.6 Balance equations for extensive quantities

The balance equations account for the changes in extensive quantities due to the inhomogeneities in their associated intensive quantities and the production or destruction of them. As we described in the introduction, the principle the balance equations is based on very simple concept; it essentially involves the bookkeeping of the extensive quantities.

#### 3.6.1 Integral form of balance equations

The integral form describes global changes in the system changes over time. It is applicable when the system of interest is homogeneous, or when local changes in the physical parameters of the system are not of concern. The *integral form* of the balance equation is more fundamental than the differential form; it has a direct physical meaning. This form is closely related to the book keeping process of extensive quantities, as it will be demonstrated in this section.

Consider an extensive quantity *X* within a control volume *V*, assumed to be compact with a closed surrounding surface *A*. The rate of change of *X* over time, *dX*(*t*)*/dt*, depends on *S*(*t*), the rate of net source of *X* within the control volume *V* at time *t*, and by *F*(*t*), the net flow rate of *X* - the net amount of *X* passing through *A* in a time interval of *dt* at time *t*. This net flow rate, *F*(*t*) = *F*_*out*_(*t*) − *F*_*in*_(*t*), is the difference between the outflow rate, *F*_*out*_, and the inflow rate, *F*_*in*_, through the volume *V* across *A*. With this interpretation of the above quantities, the integral form of the balance equation for a scalar extensive quantity, X = *X*, or for the *i*th component of a vector extensive quantity, X = *X*_*i*_, can be expressed as

(34) $$\frac{dX\left(t\right)}{dt}=S\left(t\right)-F\left(t\right)\text{,}$$

where the source terms are S (*t*) = *S*(*t*) or S (*t*) = *S*_*i*_ and the flow rates are F (*t*) = *F*(*t*) or F (*t*) = *F*_*i*_(*t*) for scalar (*X*), or for vector (**X**), extensive quantities. If the source terms and flow rates of X are zero, X is considered to be a conserved extensive quantity (*d* X*/dt* = 0). In this case, X is either a conserved scalar quantity (X = *X*) or the *i*th component (X = *X*_*i*_) of a conserved vector quantity, **X**.

If it is possible to introduce the densities of the extensive quantities and their sources, these densities can be defined implicitly by their volume integrals as

(35) $$X\left(t\right)=\int_{V}\rho_{X}\left(r,t\right)dV\text{,}$$

and

(36) $$S\left(t\right)=\int_{V}s_{X}\left(r,t\right)dV\text{,}$$

over the control volume *V*.

If vector or tensor flux densities, **j** or **J**, can be defined, the flow rates, F (*t*), of their associated scalar or vector extensive quantities become closed surface integrals of the scalar flux densities, J (**r**,*t*) = **j**(**r**,*t*), or the flux densities of the *i*th components of vector extensive quantities, J (**r**,*t*) = **J**_**i**_(**r**,*t*) over *∂V*, a closed surface containing the control volume (an application of Equation (31)),

(37) $$F\left(t\right)=\oint_{\partial V}\;J\left(r,t\right)\cdot \;\mathrm{dA,}$$

where the differential surface area vector, **dA**, points outward from the enclosed volume, ensuring that the outgoing flux is positive, in accordance with convention. Note that J (**r**,*t*) is a vector quantity and the dot product represents scalar multiplication of two vector quantities. If the densities $\rho_{X}$ (**r**,*t*), $s_{X}$ (**r**,*t*), and J (**r**,*t*) can be defined, then Equation (34) can be reformulated as

(38) $$\frac{d}{dt}\left[\;\int_{V}\rho_{X}\left(r,t\right)dV\right]=\int_{V}s_{X}\left(r,t\right)\;dV-\oint_{\partial V}\;J\left(r,t\right)\cdot \mathrm{dA,}$$

which is the *integral balance equation* expressed in densities. In this context, the integral balance equation for vector extensive quantities can be expressed, in vector notation, as

(39) $$\frac{d}{dt}\left[\;\int_{V}\rho_{X}\left(r,t\right)dV\right]=\int_{V}s_{X}\left(r,t\right)dV-\oint_{\partial V}\;J\left(r,t\right)\mathrm{dA,}$$

where the densities and flow rates are defined in Equations (35), (36), (37). Note that the multiplication of the flux density tensor, **J**, with a surface element vector **dA** is given by Equation (33).

#### 3.6.2 Differential form of balance equations

In practical applications, we often deal with inhomogeneous systems. In this case, we need to apply the *differential form* of the balance equation that describes how the system variables evolve as a function of time and position within an inhomogeneous system.

We can derive the differential form of the generic balance equation from the integral form (Equation (38)). We convert the surface integral of the flux densities to volume integral using Gauss’ theorem for the flow rates (fluxes),

(40) $$F\left(t\right)=\oint_{\partial V}\;J\left(r,t\right)\cdot \mathrm{dA}=\int_{V}\mathrm{div}J\left(r,t\right)\;dV\text{.}$$

Since we are using an Eulerian control volume, *V*, which does not change over time, the differential operator *d/dt* can be replaced by ∂/∂t in this equation. Assuming that $\rho_{X}$ (**r**, *t*) is a continuously differentiable function, therefore the differential and integral operators acting on it commute. With this assumption and using Equation (40), from Equation (38) we obtain,

(41) $$\int_{V}\left[\frac{\partial }{\partial t}\rho_{X}\left(r,t\right)+\mathrm{div}\;J\left(r,t\right)-s_{X}\left(r,t\right)\right]dV=0\text{.}$$

The choice of the control volume, *V*, is arbitrary. Therefore, Equation (41) is satisfied if the expression within the square brackets is zero. Thus, we derive the differential form of the generic balance equation for scalar extensive quantities,

(42) $$\frac{\partial }{\partial t}\rho_{X}\left(r,t\right)+\mathrm{div}\;j\left(r,t\right)=s_{X}\left(r,t\right)\text{,}$$

and for vector extensive quantities,

(43) $$\frac{\partial }{\partial t}\rho_{X}\left(r,t\right)+\mathrm{Div}\;J\left(r,t\right)=s_{X}\left(r,t\right)\text{,}$$

in component notation the *i*th component can be expressed as

(44) $$\frac{\partial }{\partial t}{\left(\rho_{X}\right)}_{i}\left(r,t\right)+\sum_{j}\frac{\partial J_{ij}}{\partial x_{j}}={\left(s_{X}\right)}_{i}\left(r,t\right)\text{.}$$

The function space that satisfies Equation (41) is broader than that which satisfies Equation (42). For the integral in Equation (41) to be meaningful, the densities must be continuous (except on a set of measure zero volume), while for solving Equation (42), the densities to be continuous and differentiable. A practical example of this distinction can be observed in shocks in fluid dynamics. Shocks can be described by functions that exhibit a jump at the shock front, which can satisfy Equation (41) but not Equation (42). This is the reason why the Rankine-Hugoniot shock jump conditions are derived using the integral balance equations.

#### 3.6.3 Balance equations for multiple extensive quantities

Assuming that the system is characterized by *N* different intensive quantities, changes in *ρ*_*k*_ (*k =* 1, …, *N*), the density of the *k*th scalar extensive quantity *X*_*k*_, are due to variations in the conjugate intensive quantities, *y*_*k*_. These changes result from matter streaming into (or out of) a control volume, conduction, and/or source and sink terms of *y*_*k*_. The balance equation for this system can be expressed as

(45) $$\frac{\partial \rho_{k}\left(r,t\right)}{\partial t}+\mathrm{div}\;j_{k}\left(r,t\right)=s_{k}\left(r,t\right)\text{,}$$

where *ρ*_*k*_, *s*_*k*_, and **j**_*k*_ represent the density, the source term, and the current density of the *k*th extensive quantity of *X*_*k*_. To simplify notation and where it does not cause any confusion, we omit the explicit dependence of these quantities on **r** and *t*. The total flux density of the *k*th intensive quantity can be expressed as

(46) $$j_{k}=\rho_{k}v_{k}+\sum_{l=1}^{N}\;L_{kl\;}\text{grad}\;\left(y_{l\;}\right)\text{,}$$

where **v**_*k*_(**r**,*t*) is the velocity of the flow of the extensive quantity, *X*_*k*_. The transport coefficients, $L_{kl}$ (**r**,*t*), (where *k* and l = 1, 2, or 3) describe the conduction due to the intensive parameter $y_{k\;}$ by the l th intensive quantity, $y_{l}$. The $L_{kl}$ (**r**,*t*) coefficients for *k* ≠ l are cross terms introduced by Onsager. Onsager's reciprocity relation states that the $L_{kl}$ coefficients are symmetric, $L_{kl}$ = $L_{lk\;}$ [52,53]. The reason for this symmetry is the time reversal invariance of the microscopic processes causing these transport phenomena (e.g., Ref. [54]). Most frequently, we have *k* = l (e.g., in diffusion, heat conduction; Sections 9.1, 9.2).

### 3.7 Balance equations and hyperbolic, parabolic, and elliptic PDEs

The balance equations are second order PDEs. We use the classification of second order PDEs to outline prescriptions for PINNs in each of the several cases (Section 11**).** These PDEs are classified as hyperbolic, parabolic, and elliptic, based on how their solutions look like in some simple cases (see Appendix B.). In PINNs, we need algorithms to solve only these three types of PDEs. The balance equation framework provides a unified algorithm to set up the relevant PDEs and the corresponding constitutive relations.

The balance equation approach starts with the principle of balance of conservation laws. In general, the dynamical form for any balanced system, the generic balance equation for scalar extensive quantity (Equation (42)) for a scalar function (or one component of a vector field) can be expressed as

(47) $$\frac{\partial u}{\partial t}+\mathrm{div}\;j=s\text{.}$$

For simplicity and clarity, we restrict our analysis to one dimension (1D). Consider the following flux (provided by a constitutive relation), and source function:

(48) $$j\left(x,t\right)=\left(\frac{1}{2\rho }\right)u^{2}\left(x,t\right)-\left(\frac{\mu }{\rho }\right)\frac{\partial u}{\partial x}+\frac{p}{\rho }\text{,}$$

where *p* (x,t) is the pressure a scalar function, and the pressure, ρ, and the dynamical viscosity, μ, are constants, and source function

(49) $$s\left(x,t\right)=\frac{f}{\rho }\text{,}$$

where *f* (x,t) the external (body) force per mass, a scalar function. In 1D, the differential operator div becomes, ∂/∂x the differential Applying the differential operator, ∂/∂x, on the flux and substituting the flux and the source functions into Equation (47), we obtain the balance equation for *u* (x,t),

(50) $$\rho \left(\frac{\partial u}{\partial t}+u\;\frac{\partial u}{\partial x}\right)=\mu \frac{\partial^{2}u}{\partial x^{2}}-\frac{\partial p}{\partial x}+f\text{,}$$

where $\partial_{t}u+u\partial_{x}u$ is the *material derivative*, the *advection* term, $u\partial_{x}u\text{,}$ coming from the convection part of the flux (first term in Equation (48)), $\mu \partial_{xx}u$ is the viscosity term from the conduction part of the flux (second term in Equation (48)), and $\partial_{x}p$ is the *pressure gradient*, and $\mu \partial_{xx}u$ is the viscosity term. We recognize, of course, the *Navier-Stokes equation* for an *incompressible flow*, and identify *u* (x,t) with v_x_ (x,t).

The Navier-Stokes equation can be either hyperbolic or parabolic. It is *hyperbolic* when *advection dominated* and *parabolic* when *viscosity dominated*.

Consider a system, that satisfies Equation (47) in 1D with the following flux (provided by the constitutive relation)

(51) j(x,t)=−k∇u(q,t),

where *j* (x,t) represents the heat flux density, k it the heat conductivity, and ∇u(q,t) is the temperature gradient, and source function

(52) s(x,t)=f(x,t),

where f(x,t) the source function for heat. In this case, our general balance equation then becomes a *parabolic* PDE:

(53) $$\frac{\partial u}{\partial t}-k\frac{\partial^{2}u}{\partial x^{2}}=f\left(x,t\right)\text{,}$$

i.e., the diffusion equation.

*Elliptic* PDEs in physics are typically parabolic systems without time dependence, i.e., the parabolic system, Equation (53), becomes

(54) $$\frac{\partial^{2}u}{\partial x^{2}}=g\left(x,t\right)\text{,}$$

where g(x,t)=f(x,t)/k, and we recognize Poisson's equation. In physical applications, u(x,t), and f(x,t) could be the electric field the electric charge.

## 4 Continuous systems

Almost all classical systems can be modeled using continuous fields. In most applications we can assume that the densities of all extensive and intensive quantities are continuous. While not always the case, continuum thermodynamics is often included in discussions about continuous systems. This approach assumes local thermodynamic equilibrium (LTE), which is a reasonable approximation if the thermodynamic variables (e.g., temperature, pressure, entropy) can be defined at nearly every point within a system. The most common materials, including solids (e.g., metals, alloys, plastic, etc.) and fluids (e.g., liquids and gases), are typically described as continuous systems. In applications of continuous systems, the differential form of balance equations must be used for analyzing local changes in system variables. This is why we focus on this form in our discussion.

In general, any system represented by continuous fields can be described by generic balance equations. The most important extensive quantities for a physical system are total mass, momentum, and energy. The balance equations for these quantities become conservation equations once we take into account their respective sources and sinks. Thus, the total mass, momentum, and energy conservation equations are the most important balance equations of continuous systems. In the absence of sources or sinks for these quantities, the balance equations for mass, momentum, and energy express conservation laws. In most applications, the differential form of their balance equations is used, assuming that the continuous fields are also differentiable.

Depending on the applications, the inhomogeneities of various intensive quantities generate different fluxes of their associated extensive quantities. These quantities are referred to as the relevant extensive and intensive quantities for a given system.

### 4.1 Equation of total mass conservation

We can derive the mass conservation equation from the generic balance equation for scalar extensive quantities, Equation (42), by identifying *ρ*_*X*_(**r**,*t*) as the total mass density of *N* different species, *ρ*_*X*_ (r,t) = $\rho \left(r,t\right)=\sum_{k}^{N}\rho_{k}\left(r,t\right)\text{,}$ and using $j=\rho v+\sum_{k}^{N}L_{Dk}\nabla \mu_{k}$ for the flux. Here ρ(r,t), $L_{Dk}\left(r,t\right)$, and $\mu_{k}\left(r,t\right)$, and are the density, the conduction coefficient (related to diffusion), and the chemical potential of species *k* (*k* = 1, 2, …, *N*), and v(r,t) is the velocity of the flow carrying ρ, assumed to be the same for all species. Assuming that no sources exist for the total mass, we use *s*_*X*_ (r,t) = 0 (this assumption is satisfied in classical systems). With these assumptions, we obtain the mass conservation equation from Equation (42) as

(55) $$\frac{\partial \rho }{\partial t}+\mathrm{div}\;\left[\rho \;v\;\right]+\sum_{k=1}^{N}\mathrm{div}\;\left[L_{Dk}\nabla \mu_{k}\;\right]=0\text{.}$$

### 4.2 Equation of momentum conservation

The momentum associated with the flow of a scalar extensive quantity, the total mass, is a vectorial extensive quantity. The momentum density can be expressed as **ρ**_**p**_(**r**,*t*) = ρ (**r**,*t*) **v**(**r**,*t*), where ρ (**r**,*t*) is the mass density and v(r,t) is the velocity of the flow.

The momentum flux density of a vector intensive quantity, the momentum, is a second-rank tensor, **J**(**r**,*t*). **J**(**r**,*t*) is a sum of convective and conductive terms. The conductive term is generated by momentum transfer due to stress (force over infinitesimal area) within a continuous body at position **r** and time *t*. This stress is described by a second-rank tensor, the Cauchy stress tensor, **σ**(**r**,*t*). The diagonal terms of the Cauchy stress tensor, *σ*_xx_, *σ*_yy_, and *σ*_zz_, describe tension generated by contact forces at position **r** and time *t* over infinitesimal surfaces *dydz*, *dxdz*, *and dxdy* normal to the *x*, *y*, and *z* directions. In general, the stress vector or surface traction, **t**_**n**_(**r**,*t*), at a chosen differential area is defined as **t**_**n**_(**r**,*t*) = **n σ**(**r**,*t*), where **n** is a unit normal vector to the differential area. In component notation, *t*_*j*_(**r**,*t*) = *n*_*i*_*σ*_*ij*_(**r**,*t*). The form of the Cauchy stress tensor depends on the application (e.g., fluid dynamics, elasticity; see Sections 5, 6). Thus, using Equations (14), (17) with **ρ**_**X**_(**r**,*t*) = *ρ*(**r**,*t*) **v**(**r**,*t*) and **J**_cd_(**r**,*t*) = –**σ** (**r**,*t*), we obtain the momentum flux density tensor,

(56) J(r,t)=ρ(r,t)v(r,t)⊗v(r,t)−σ(r,t).

We derive the differential form of the momentum balance equation from Equation (43) by setting ***ρ***_***X***_(**r**,*t*) = *ρ*_*X*_(**r**,*t*)**v**(**r**,*t*) and **s**_*X*_(**r**,*t*) = **f**(**r**,*t*), where **f**(**r**,*t*) is the external body force (a force density acting on the body at position **r** and time *t*), and using the momentum flux density (Equation (56)),

(57) $$\frac{\partial \rho v}{\partial t}+\mathrm{Div}\left[\rho \;v⊗v-\sigma \right]=f\text{;}$$

in component notation, the *i*th component is

(58) $$\frac{\partial \rho v_{i}}{\partial t}+\sum_{j}\frac{\partial }{\partial x_{j}}\left[\rho v_{j}v_{i}-\sigma_{ij}\right]=f_{i}\text{.}$$

This equation represents *Cauchy's first law of motion* in continuum mechanics.

The conservation of angular momentum leads to the constraint that the Cauchy stress tensor must be symmetric (**σ** is equal to its transpose, **σ** = **σ** ^T^), which constitutes *Cauchy's second law of motion*.

In general, the Cauchy stress tensor may originate from inhomogeneities in the relevant intensive quantities, **y**(**r**,*t*) (e.g., elasticity, see Section 6.2), and from normal stresses due to thermodynamic pressure, *p* (e.g., in fluids),

(59) $$\sigma \left(r,t\right)=\sigma_{y}\left(r,t\right)+\sigma_{p}\left(r,t\right)\text{,}$$

where

(60) $$\sigma_{p}\left(r,t\right)=-p\left(r,t\right)\;I_{3}\text{.}$$

Note that while **σ**_**y**_ depends on the properties of matter (the conduction coefficients), **σ**_p_ is independent of them, since the thermodynamic pressure originates directly from kinematics: the random motion of particles.

The Cauchy stress tensor, **σ**, can be decomposed as the sum of a deviatoric stress tensor, **τ**, and a volumetric stress or mean stress, π,

(61) $$\sigma \left(r,t\right)=\tau \left(r,t\right)+\pi \left(r,t\right)\;I_{3}\text{;}$$

in component notation,

(62) $$\sigma_{ij}=\tau_{ij}+\pi \;\delta_{ij}\text{,}$$

where

(63) $$\pi =\frac{1}{3}\sum_{k=1}^{3}\sigma_{kk}\;\text{.}$$

The traceless deviatoric stress tensor, Trace{**τ**} = 0, describes shear. The volumetric stress, *π*, causes pure volume expansion or contraction.

The physical interpretation of **τ** and *π* depend on the applications. In non-viscous fluids in equilibrium, for example, the thermodynamic pressure, *p*, causes normal isotropic stresses in a fluid, but no shear stresses, thus **τ** = 0, and the mean stress is equal to the negative of the thermodynamic pressure, *π* = −*p*. However, in some applications, e.g., describing solids, –*π* is called the mechanical pressure, *p*_*mech*_ = –*π*, but *π* may not be identified with the thermodynamic pressure (in general, *p ≠ p*_*mech*_).

In most applications, we may assume that the material is isotropic and linear. In this context, the flux density stress tensor due to conduction, **σ**_**y**_, can be described by two parameters, e.g., *L*_1_ and *L*_2_ (Equation (26) with **J**_cd_ = –**σ**_**y**_; refer to Section 3.5.1).

In continuum mechanics, a frequently used parameterization for linear isotropic materials was introduced by Lamé,

(64) $$\sigma_{y}=2\mu \;\varepsilon \left(y\right)+\lambda \;\left(\mathrm{div}\;y\right){\;I}_{3}\text{,}$$

where *λ* and *μ* are the first and second Lamé parameters (Equation (26) with *L*_*1*_ = –2*μ* and *L*_*2*_ = –*λ*), and **ε**(**y**) is the strain tensor defined in Equation (27). Thus, in linear isotropic materials, the total stress tensor (i.e., Cauchy stress tensor), Equation (59), using Equation (64), can be written as

(65) $$\sigma \left(r,t\right)=2\mu \;\varepsilon \left(y\right)+\left[\lambda \;\left(\mathrm{div}\;y\right)-p\right]\;I_{3}\text{.}$$

The Cauchy tensor depends on material properties; thus, Equation (65) is also called a constitutive relation.

### 4.3 Equation of energy conservation

We define the total energy density, *e*(**r**,*t*), as the sum of the kinetic ((1/2) *ρ* **v**^2^) and internal (*e*_*int*_) energy densities,

(66) $$e\left(r,t\right)=\frac{1}{2}\rho \left(r,t\right)\;v^{2}\left(r,t\right)+e_{\mathrm{int}}\left(r,t\right)\text{,}$$

where **v**(**r**,*t*) is the flow velocity carrying the total mass density, ρ(r,t). The total energy density is the density associated with the extensive quantity, the total energy.

In general, the source of change in the energy density is the rate of work done on the system by external body forces, **f**(**r**,*t*), and the rate at which heat is produced, *s*_*q*_(**r**,*t*). Thus, for the source term, we obtain

(67) $$s\left(r,t\right)=f\left(r,t\right)\cdot v\left(r,t\right)+s_{q}\left(r,t\right)\text{.}$$

The net energy flux density, **j**(**r**,*t*), is the sum of the convective and conductive energy transport,

(68) j(r,t)=e(r,t)v(r,t)−σ(r,t)v(r,t)+q(r,t),

in component notation, the *i*th component is

(69) $$j_{i}=ev_{i}-\sum_{j}\sigma_{ij}v_{j}+q_{i}\text{,}$$

where **σ**(**r**,*t*) is the Cauchy stress tensor (Equation (61)), **σ v** (the product of a 2nd-rank tensor with a vector defined in Equation (A1)) is the rate of the work done by tension as expressed by the Cauchy stress tensor, and **q** is the heat flux density, i.e., the rate of energy transport by heat conduction.

In linear materials, the heat flux density, **q**, can be expressed as

(70) $$q\left(r,t\right)=L_{q}\left(r,t\right)\nabla T\left(r,t\right)\text{,}$$

where **L**_**q**_ is the conduction coefficient for heat transport and *T* is the temperature of the system. In general, in anisotropic materials, ***L***_***q***_ is a 2nd-rank tensor. In component notation, its *i*th component can be expressed as

(71) $$q_{i}\left(r,t\right)=\sum_{j}{\left(L_{q}\right)}_{ij}\partial_{j}T\left(r,t\right)\text{.}$$

In linear isotropic materials the tensor conduction coefficient for heat transport takes the form (*L*_*q*_)_*ij*_ = *L*_*q*_ *δ*_*ij*_, where *L*_*q*_(**r**,*t*) is a scalar function. Therefore, the heat flux can be expressed as

(72) $$q\left(r,t\right)=L_{q}\left(r,t\right)\nabla T\left(r,t\right)\text{.}$$

We obtain the differential form of the balance equation for the total energy density, *e*(**r**,*t*), from the generic balance equation, Equation (42), by applying Equations (66), (67), (68),

(73) $$\frac{\partial e}{\partial t}+\mathrm{div}\;\left[e\;v-\sigma \;v+q\right]=f\cdot v+s_{q}\text{,}$$

in component notation,

(74) $$\frac{\partial e}{\partial t}+\sum_{ij}\partial_{i}\left[ev_{i}-\sigma_{ij}v_{j}+q_{i}\right]=\sum_{i}f_{i}v_{i}+s_{q}\text{.}$$

Thermodynamic processes change the internal energy of a system. We derive the balance equation for the internal energy density, *e*_*int*_(**r**,*t*), by taking the scalar product of the balance equation for momentum density with the velocity vector, (**v** · Equation (57)), subtracting the result from Equation (73), and applying Equation (66),

(75) $$\frac{\partial e_{\mathrm{int}}}{\partial t}+\mathrm{div}\;\left(e_{\mathrm{int}}\;v+q\right)=\sigma :ɛ\left(v\right)+s_{q}\text{,}$$

where the double-dot notation stands for the scalar product of two tensors of the same rank, in this case, $\sigma :\varepsilon =\sum_{ij}\sigma_{ij}\varepsilon_{ij}$ (Equation (A8)). This equation describes how the internal energy changes over time due to thermodynamic and mechanical processes.

### 4.4 Summary of continuous systems

Continuous systems can be described by the conservation equations for total mass, momentum, and energy. We derived these equations from the generic balance equations. Once the sources and fluxes for the extensive quantities, such as mass, momentum, and energy, are determined, their conservation equations can be derived. We have summarized the relevant sources and fluxes in Table 4. The fluxes shown are based on the most commonly used approximation, the linear isotropic approximation. In this approximation, the generalized strain tensor, ε(y), is given by Equation (27). Note that the vector field, the intensive quantity **y**, just like the other quantities, is local, and it should be evaluated at position **r**, and time *t* (y=y(r,t)). The interpretation of scalar (e.g., *λ* (r,t) and *μ* (r,t)) and vector fields (ε(y(r,t)) depend on the applications, (e.g., fluid dynamics and elasticity; see Sections 5, 6).

**Table 4:** Relevant extensive quantities of continuum systems and their sources and fluxes (linear isotropic approximation).

Relevant extensive quantity	Source	Flux densities (convective + conductive)	
		Convective	Conductive (linear, isotropic)
Mass [ρ(r,t)]	0	*ρ*(r,t)v(r,t)	$\sum_{i}L_{Di}\left(r,t\right)\nabla \mu_{i}\left(r,t\right)$
Momentum [p(r,t)]	f(r,t)·v(r,t)	ρ(r,t)v(r,t)⊗v(r,t)	$–2\mu \left(r,t\right)\;\varepsilon \left(y\right)\;–\left[\lambda \left(r,t\right)\;\left(\mathrm{div}\;y\right)-p\left(r,t\right)\right]I_{3}$
Total energy [e(r,t)]	e(r,t)·v(r,t)	e(r,t)v(r,t)	−2με(y)v(r,t)–[λ(r,t)(divy)−p(r,t)]v(r,t)

## 5 Fluid mechanics

Fluid mechanics is a branch of continuum mechanics and thermodynamics. Fluid mechanics is extensively used to describe the physics of many systems of interest in science and engineering. In this context “fluids” means that conditions for continuum approximation is valid for the system (e.g., physical fluids, gases). In other words, it assumes that the fluid approximation if is correct, i.e., the physical processes can be described by macroscopic variables, e.g., density, pressure, temperature, ignoring the processes on atomic level.

Physical processes in fluids can be completely described by the conservation equations of mass, momentum, and total energy. In this section, we derive the basic equations of fluid mechanics based on our results for continuous systems discussed in Section 4. We will present some important applications in Section 9.

### 5.1 Mass conservation: Mass continuity equation

In fluid dynamics, assuming that the conduction coefficients for mass transport are zero (*L*_*Di*_ (r,t)=0), the mass conservation equation (Equation (55)) simplifies to the most well-known balance equation, the continuity equation for the mass density, *ρ*(**r**,*t*),

(76) $$\frac{\partial }{\partial t}\rho \left(r,t\right)+\mathrm{div}\left[\rho \left(r,t\right)\;v\left(r,t\right)\right]=0\text{.}$$

The mass continuity equation describes the flow of the conserved extensive quantity, the mass, within a system. It asserts that the change in mass over time within an infinitesimal volume around a fixed point must equal to the difference between the mass inflow and outflow through the closed boundary of the volume.

### 5.2 Momentum conservation: Navier–Stokes equation

We derived the momentum equation for a continuous system in Section 4.2. Equation (57) of that section will serve as the basis for our derivation of the momentum equation of fluid dynamics, using the expressions for the Cauchy stress tensor provided in Equations (64), (65).

In fluid dynamics, the momentum flux density due to conduction, **J**_cd_(**r**,*t*), is described by the Cauchy stress tensor, **σ**: **J**_cd_(**r**,*t*) = –**σ**(**r**,*t*). Conduction may be originated from velocity gradients in the fluid generated by and the thermodynamic pressure, *p*(**r**,*t*), due to thermal motion of fluid particles:

(77) $$\sigma \left(r,t\right)=\sigma_{v}\left(r,t\right)+\sigma_{p}\left(r,t\right)\text{,}$$

where **σ**_v_(**r**,*t*) is the viscous stress tensor and **σ**_p_(**r**,*t*) is the pressure stress tensor, **σ**_p_(**r**,*t*) = –*p*(**r**,*t*)**I**_3_ (Equation (60)).

The momentum density flux, described by the viscous stress tensor, **σ**_v_(**r**,*t*), originates from the spatial inhomogeneities in the velocity fields of the fluid. Thus, the fluid velocity, **v**(**r**,*t*), takes the role of an intensive vector variable, **y**(**r**,*t*) = **v**(**r**,*t*), associated with the extensive quantity, the momentum. Note that the pressure is also related to the inhomogeneities in the velocity fields, specifically to the velocity dispersion of the local equilibrium distribution of the particle velocities.

In most applications of fluid dynamics, we may assume that the dependence of the viscous stress tensor, **σ**_v_(**r**,*t*), on the derivatives of the associated intensive variable, the velocity, **v**(**r**,*t*), is linear. This type of fluids is are termed Newtonian fluid. Thus, for a Newtonian fluid, we can use our results for linear materials we derived in Section 3.5.1. Using the relevant intensive variable, the velocity, **y**(**r**,*t*) = **v**(**r**,*t*), in the expression for the generalized strain tensor, Equation (27), we obtain

(78) $$\varepsilon \left(v\right)=\frac{1}{2}\left[\text{Grad}\;v+{\left(\text{Grad}\;v\right)}^{T}\right]\text{;}$$

in component notation,

(79) $$\varepsilon_{ij}=\frac{1}{2}\left[\frac{\partial v_{i}}{\partial x_{j}}+\frac{\partial v_{j}}{\partial x_{i}}\right]\text{.}$$

In fluid dynamics, **ε**(**v**) is referred to as the *rate of strain tensor*, as it describes the rate of deformation of fluid cells.

In isotropic Newtonian fluids, the viscous stress tensor, **σ**_v_, can be parameterized using the first and second Lamé parameters, *λ* and *μ*, as in continuum mechanics (see Equation (64), and the explanation in Section 3.5.1). In this context, *λ* and *μ*, are also known as *bulk* and *dynamic viscosity*.

In fluid dynamics, it is customary to separate the traceless deviatoric stress and volumetric (mean) stress components of the viscous stress tensor, and write $\sigma_{v}$ as (Equation (64) with **y**(**r**,*t*) = **v**(**r**,*t*))

(80) $$\sigma_{v}=2\mu \left[\varepsilon \left(v\right)-\frac{1}{3}\left(\mathrm{div}\;v\right)\;I_{3}\right]+\zeta \;\left[\mathrm{div}\;v\right]\;I_{3}\text{,}$$

where

(81) $$\zeta =\lambda +\frac{2\mu }{3}$$

is the *second viscosity coefficient*. The viscous stress tensor (Equation (80)) depends on material variables, thus, Equation (80) is also called a constitutive relation for fluid dynamics. Note that the viscosity coefficients generally depend on position and time: *μ* = *μ* (**r**,*t*) and *ζ* = *ζ* (**r**,*t*). With this parameterization (Equations (77), (80)), the Cauchy stress tensor (Equation (61)) in fluid dynamics becomes

(82) $$\sigma =2\mu \left[\varepsilon \left(v\right)-\frac{1}{3}\left(\mathrm{div}\;v\right)\;I_{3}\right]+\left[\zeta \;\mathrm{div}\;v-p\right]\;I_{3}\text{,}$$

suggesting that, in fluid dynamics,

(83) $$\tau =2\mu \left[\varepsilon -\frac{1}{3}\left(\mathrm{div}\;v\right)\;I_{3}\right]\text{,}$$

and

(84) π=ζdivv−p.

Note, that in the case of constant second viscosity, *ζ*, an effective pressure can be introduced,

(85) $$p_{eff}=-\pi =p-\zeta \;\mathrm{div}\;v\text{.}$$

In this simple case, the momentum flux density tensor, Equation (82), becomes

(86) $$J=\rho \;v⊗v-\tau +p_{eff}\;I_{3}\text{,}$$

where **τ** and *p*_*eff*_ are given in Equations (82), (85).

In linear materials, assuming that *μ* and *ζ* are homogeneous (∇ *μ =* ∇ *ζ =* 0) and carrying out the Div differential operator on the Cauchy tensor, **σ** (Equation (82)), we obtain

(87) $$\mathrm{Div}\;\sigma =\mu \;\Delta v+\left[\zeta +\frac{1}{3}\mu \;\right]\nabla \left(\mathrm{div}\;v\right)-\nabla p\text{.}$$

In component notation, the *i*th component can be expressed as

(88) $$\sum_{j}\frac{\partial \sigma_{ij}}{\partial x_{j}}=\mu \;\sum_{k}{\left(\partial_{k}\right)}^{2}{\;v}_{i}+\left[\zeta +\frac{1}{3}\mu \right]\partial_{i}\left(\sum_{k}\partial_{k}v_{k}\right)-\nabla p\text{,}$$

where Δ**v** is the Laplace operator acting on a vector field, **v**(**r**,*t*) (Equation (A6)). With these assumptions and parameterization, and applying Equation (87), from Equation (57) we derive the balance equation for the momentum in an isotropic and homogeneous material (∇ *μ =* ∇ *ζ =* 0), we obtain

(89) $$\frac{\partial \rho v}{\partial t}+\mathrm{Div}\left[\rho \;v⊗v\right]=\mu \;\Delta v+\left[\zeta +\frac{1}{3}\mu \;\right]\nabla \left(\mathrm{div}\;v\right)-\nabla p+f\text{.}$$

Equation (89) is known as the *conservation form* of the Navier–Stokes equations.

The *convective* form of the Navier–Stokes equations, which is more frequently used, can be derived from Equation (89) by applying the tensor differential operator, Div, to the convective term,

(90) Div[ρv⊗v]=ρv(divv)+(v·∇)(ρv),

and applying the continuity equation, Equation (76), we obtain

(91) $$\rho \left[\frac{\partial v}{\partial t}+\left(v\cdot \nabla \right)v\right]=\mu \;\Delta v+\left[\zeta +\frac{1}{3}\mu \;\right]\nabla \left(\mathrm{div}\;v\right)-\nabla p+f\text{;}$$

in component notation, the *i*th component is

(92) $$\rho \left[\frac{\partial v_{i}}{\partial t}+\sum_{k}\left(v_{k}\partial_{k}\right)v_{i}\right]=\mu \sum_{k}\partial_{k}^{2}v_{i}+\left[\zeta +\frac{1}{3}\mu \;\right]\partial_{i}\sum_{k}\left(\partial_{k}v_{k}\right)-\partial_{i}p+f_{i}\text{.}$$

In some applications, *body acceleration*, **a**(**r**,*t*), is used in place of force density. In such cases **f**(**r**,*t*) = *ρ*(**r**,*t*) **a**(**r**,*t*). For example, if gravity is the only external force, then **f**(**r**,*t*) = *ρ*(**r**,*t*) **g**(**r**,*t*), where **g**(**r**,*t*) is the gravitational acceleration.

In the context of a system consisting of monatomic fluid, the second viscosity coefficient becomes zero, *ζ* = 0 (Stokes hypothesis), and the Navier–Stokes equations become

(93) $$\rho \left[\frac{\partial v}{\partial t}+\left(v\cdot \nabla \right)v\right]=\mu \;\Delta v+\left(1/3\right)\mu \nabla \left(\mathrm{div}\;v\right)-\nabla p+f\text{.}$$

Another frequently used special case is when the fluid can be considered incompressible, div **v** = 0. In this case the Navier–Stokes equation simplifies to

(94) $$\frac{\partial v}{\partial t}+\left(v\cdot \nabla \right)\;v=\nu \;\Delta v-\frac{1}{\rho }\nabla p+a\text{,}$$

where we introduced the *kinematic viscosity*, ν = *μ/ρ*, and used the body acceleration, **a** = **f***/ρ*. The assumption of incompressible flow is typically applicable to fluids or gases (e.g., air) when the velocities are less than about 0.3 of the sound speed, *c*_*s*_, in the fluid (Mach number, *M =* v*/c*_*s*_ < 0.3).

### 5.3 Energy equation of fluid dynamics

We derive the energy equation for fluid dynamics from Equation (73), the balance equation for the total energy density, *e =* ½ *ρ*v^*2*^*+e*_*int*_, using the Cauchy stress tensor of fluid dynamics (Equation (82)),

(95) $$\frac{\partial e}{\partial t}+\mathrm{div}\;\left[e\;v-\sigma \;v+q\right]=f\cdot v+s_{q}\text{,}$$

where, **q**(**r**,*t*) is the heat flow. In linear materials it is given by Equation (70).

Applying the differential operator to (**σ v**) in Equation (95), we obtain

(96) $$\frac{\partial e}{\partial t}+\mathrm{div}\;\left(e\;v+q\right)-\left(\mathrm{Div}\;\sigma \right)\cdot v-\sigma :\varepsilon \left(v\right)=f\cdot v+s_{q}\text{,}$$

where $\sigma :\varepsilon =\sum_{ij}\sigma_{ij}\varepsilon_{ij}$ (Equation (A8)). This important term in thermodynamics of continuous media is known as the *stress power*.

In homogeneous and isotropic materials, the Lamé parameters and the heat conduction coefficient are independent of position (∇ *μ =* ∇ *λ =* ∇ *L*_*q*_ = 0). Thus, in this case, by using the Cauchy stress tensor and its divergence (Equations (65), (87)), we obtain

(97) $$\left(\mathrm{Div}\;\sigma \right)\cdot v=\mu \;v\cdot \Delta y+\left[\zeta +\frac{1}{3}\mu \;\right]v\cdot \nabla \left(\mathrm{div}\;y\right)-v\cdot \nabla p\text{,}$$

and

(98) $$\sigma :\varepsilon \left(v\right)=2\mu \;\varepsilon \left(v\right):\varepsilon \left(v\right)+\left[\zeta -\left(2/3\right)\mu \right]\;{\left(\mathrm{div}\;v\right)}^{2}-p\;\left(\mathrm{div}\;v\right)-L_{q}\;\Delta T+f\cdot v+s_{q}\text{.}$$

We derive the energy equation of fluid dynamics in linear isotropic materials from Equation (96) using Equations (97), (98), and, for the heat flux, Equation (72), assuming that *μ, ζ*, and *L*_*q*_ are homogeneous (∇*μ* = ∇*ζ* = ∇*L*_*q*_ = 0),

(99) $$\begin{array}{ll} \;\frac{\partial e}{\partial t}+\mathrm{div}\;\left(e\;v\right) & =\mu \;v\cdot \Delta v+\left[\zeta +\left(1/3\right)\mu \right]v\cdot \nabla \left(\mathrm{div}\;v\right)-v\cdot \nabla p+2\mu \;\varepsilon \left(v\right):\varepsilon \left(v\right)+\left[\zeta -\left(2/3\right)\mu \right]\;{\left(\mathrm{div}\;v\right)}^{2}\\ & -p\;\left(\mathrm{div}\;v\right)-L_{q}\;\Delta T+f\cdot v+s_{q} \end{array}$$

Thermodynamic processes change the internal energy of the fluid. We derive the balance equation for internal energy density in a fluid from Equation (75), using the Cauchy stress tensor, **σ** (Equation (82)), and, as it is common in most applications, neglecting the heat flux production (*s*_*q*_ = 0),

(100) $$\frac{\partial }{\partial t}e_{\mathrm{int}}+\mathrm{div}\;\left(e_{\mathrm{int}}\;v+q\right)=\sigma :\varepsilon \left(v\right)\text{.}$$

In this case, assuming linear isotropic fluid (*μ, ζ*, and *L*_*q*_ are homogeneous), we obtain

(101) $$\frac{\partial e_{\mathrm{int}}}{\partial t}+\mathrm{div}\;\left(e_{\mathrm{int}}\;v\right)+L_{q}\;\Delta T=2\mu \;\varepsilon \left(v\right):\varepsilon \left(v\right)+\left[\zeta -\left(2/3\right)\mu \right]\;{\left(\mathrm{div}\;v\right)}^{2}-p\;\left(\mathrm{div}\;v\right)\text{.}$$

### 5.4 Entropy equation

Entropy is conserved only in reversible processes. However, by including the internal entropy production in the entropy balance, we can obtain a balance equation for the specific entropy density.

The balance equations for entropy density, *s*(**r**,*t*), can be derived from Equation (42) by setting *ρ*_*X*_(**r**,*t*) = *s*(**r**,*t*), we obtain

(102) $$\frac{\partial s}{\partial t}+\mathrm{div}\;\left[s\;v-\frac{q}{T}\right]=\frac{r}{T}+s_{\mathrm{int}}\text{,}$$

where the entropy flux density (**j**) comprises two terms: a convective term, *s***v** and a conductive term, **q**/*T*, i.e., the outgoing heat flux vector over temperature (note that Δ*q* = *T*Δ*s*). The source terms on the right hand side are generated by an external heat source density, *r*(**r**,*t*), and the internal entropy production, *s*_*int*_(**r**,*t*)*.* Note that if we do not take into account the internal entropy generation (setting *s*_*int*_ = 0), we have only the Clausius-Duhem inequality,

(103) $$\frac{\partial s}{\partial t}+\mathrm{div}\;\left[s\;v-\frac{q}{T}\right]\geq \frac{r}{T}\text{,}$$

which is a consequence of the Second Law of thermodynamics.

## 6 Elasticity

Elasticity is a branch of continuum mechanics that describes how an elastic body deforms under stresses due to external forces. Many different objects around us (e.g., metal beams, bridges, buildings) can be modeled by elasticity, making it a significant area of application of continuum mechanics in science and engineering.

In our treatment of elasticity, we employ the displacement formulation in our treatment of elasticity. In this approach, the deformation of the elastic body is characterized by the displacement vector field, **u**(**r**,*t*). It is defined at every point, *P*, in the body as the difference between the new position, **r**_P_ (*t*), of point *P* at time *t* and **r**_P_ (*t*) and the original position, **r**_P_(0): **u**(**r**,*t*) = **r**_P_(*t*) - **r**_P_(0).

If all the points in the elastic body are displaced by the same vector, it is clear that no tension arises within the body. Tension is caused by inhomogeneities in the displacement vector field. When the force causing the deformation is removed, all the displaced points in the elastic body return to their equilibrium position (note that this is the definition of an elastic body), resulting in momentum transfer. This momentum transfer is described by the Cauchy stress tensor. In elasticity, the velocity of the deformation, which describes the rate of distortion of the body, is defined as the time derivative of the displacement vector: **v**(**r**,*t*) = ∂**u**/∂t. Thus, the displacement vector, **u**(**r**,*t*), serves as the *intensive quantity* in elasticity, with its associated extensive quantity being the momentum density, **p**(**r**,*t*) = *ρ*(**r**,*t*) **v**(**r**,*t*), where *ρ*(**r**,*t*) is the mass density.

In many applications, it is often a reasonable assumption that the deformation of the material is linearly proportional to the applied strain. Therefore, linear elasticity is widely used in structural analysis and engineering design. In this section we derive the basic equations of linear elasticity, including the conservation of momentum and energy, based on the balance equations in the displacement formulation.

### 6.1 Mass conservation: Continuity equation

In linear elasticity, we assume small (infinitely small) deformations, and thus we assume that the flow velocity is zero (*ρ*(**r**,*t*) **v**(**r**,*t*) = 0), resulting in no conductive flux. Under these assumptions, we apply the continuity equation (Equation (76)) and obtain

(104) $$\frac{\partial }{\partial t}\;\rho \left(r,t\right)=0\text{.}$$

As a consequence, we may consider the mass density to be constant over time, *ρ*(**r**,*t*) = 0, allowing us to ignore the small changes in it due to stress.

### 6.2 Momentum conservation: Navier's equations of elasticity

In elastic processes, the flow velocity is assumed to be zero since we are dealing with only distortions of elastic bodies). The velocity in elasticity, **v**(**r**,*t*), describes the rate of distortion of the body.

In linear elasticity, stress – the applied force over an area – is assumed to be linearly proportional to the resulting strain. The simplest case is the response of an elastic material to a unidirectional strain, where the stress can be calculated as *σ* = *F*/*A*, where *F* is the applied force over an area *A*. The stress is proportional to the resulting strain, *ε*, defined as the change of the length over the original length of the material (e.g., beam of steel), *ε* = Δ*L*/*L*. Accordingly, *σ* = *E* Δ*L*/*L* = *E ε*, where *E* is the *Young's modulus*, this is referred to as *Hooke's law*.

In 3D, the stress is generalized to the *strain tensor* of elasticity. The stress inside the material originates from inhomogeneities in the relevant intensive quantity of elasticity, described by the displacement vector field, **u**(**r**,*t*). Thus, in linear elasticity, we have **y**(**r**,*t*) = **u**(**r**,*t*), and the strain tensor (Equation (27)), becomes

(105) $$\varepsilon \left(u\right)=\frac{1}{2}\left[\text{Grad}\;u+{\left(\text{Grad}\;u\right)}^{T}\right]\text{,}$$

in component notation,

(106) $$\varepsilon_{ij}=\frac{1}{2}\left[\frac{\partial u_{i}}{\partial x_{j}}+\frac{\partial u_{j}}{\partial x_{i}}\right]\text{.}$$

In a linear elastic material in 3D, this momentum transfer is described by the Cauchy stress tensor in the form of a generalization of Hooke's law. The generalized Hooke's law asserts that each component of the Cauchy stress tensor, *σ*_*ij*_, depends linearly on the generated strain, as described by the strain tensor, *ε*_*ij*_,

(107) $$\sigma_{ij}\left(r,t\right)=C_{ijmn}\left(r,t\right)\;\varepsilon_{ij}\left(r,t\right)\text{,}$$

where the 4th-rank tensor, *C*_*ijmn*_ is the stiffness tensor (compare Equation (107) to Equation (24)).

In an isotropic linear material, the stiffness tensor, *C*_*ijmn*_, has only two independent parameters based on the same argument presented in Section 3.5.1. Thus, the Cauchy stress tensor can be expressed as

(108) $$\sigma \left(u\right)=2\mu \;\varepsilon \left(u\right)+\lambda \;\left(\mathrm{div}\;u\right)\;I_{3}\text{,}$$

where *λ* (r,t), and *μ* (r,t), are the first and second Lamé parameters. Since Equation (108) depends on material parameters, it is also called a constitutive relation for elasticity. The strain tensor, **ε**(**u**) is defined in Equation (105) (Equation (64) with **y** = **u**).

Assuming that the Lamé parameters, *μ* and *λ* are homogeneous (∇ *μ =* ∇ *λ =* 0) and applying the Div operator to the Cauchy tensor, **σ** (Equation (108)), we obtain

(109) Divσ=μΔu+(μ+λ)∇(divu)−∇p,

in component notation,

(110) $$\sum_{j}\frac{\partial \sigma_{ij}}{\partial x_{j}}=\mu \sum_{k}{\left(\partial_{k}\right)}^{2}u_{i}+\left(\mu +\lambda \right)\;\partial_{i}\sum_{k}\left(\partial_{k}u_{k}\right)-\nabla p\text{,}$$

where Δ**u** is the Laplace operator acting on a vector field, **u**(**r**,*t*) (Equation (A6)). With these assumptions and parameterization, and by using Equation (87), the balance equation for the momentum density in an isotropic and homogeneous material, Equation (57), in conservation form becomes

(111) $$\frac{\partial \rho v}{\partial t}+\mathrm{Div}\left[\rho \;v⊗v\right]=\mu \;\Delta u+\left(\mu +\lambda \right)\nabla \left(\mathrm{div}\;u\right)-\nabla p+f\text{.}$$

We derive the *convective* form of the momentum balance equation from Equation (111) by applying the tensor differential operator, Div, to the convective term (Equation (90)), and using the continuity equation (Equation (76)), we obtain

(112) $$\rho \left[\frac{\partial v}{\partial t}+\left(v\cdot \nabla \right)v\right]=\mu \;\Delta u+\left(\mu +\lambda \right)\nabla \left(\mathrm{div}\;u\right)-\nabla p+f\text{.}$$

Based on our interpretation and parameterization of the Cauchy stress tensor (Equation (108)), and using the displacement vector as our intensive variable, **y**(**r**,*t*) = **u**(**r**,*t*) and **v** = ∂**u**/∂t for velocity, we assume no convective transport, no pressure term (*p* = 0), no heat conduction (**q**(**r**,*t*) = 0), and homogeneous Lamé parameters (∇ *μ =* ∇ *λ =* 0). From Equation (112), we obtain the Navier's equations of elasticity,

(113) $$\rho_{0}\frac{\partial^{2}u}{\partial t^{2}}=\mu \;\Delta u+\left(\mu +\lambda \right)\nabla \left(\mathrm{div}\;u\right)+f\text{,}$$

where we used *ρ*(**r**,*t*) = *ρ*_0_(**r**,0), which is the consequence of the continuity equation of elasticity (Equation (104)).

### 6.3 Energy equation of elasticity

In linear elasticity, the kinetic energy can be ignored, and the applied strain increases the internal energy. Thus the system can be described by the internal energy density, *e*_*int*_(**r**,*t*). Assuming that the flow velocity is zero and there is no energy input (no external heat, *q*(**r**,*t*) = *s*_*q*_(**r**,*t*) = 0), from Equation (75), we derive the internal energy density balance equation for linear elasticity,

(114) $$\frac{\partial e_{\mathrm{int}}}{\partial t}=\sigma \left(u\right):\varepsilon \left(v\right)\text{;}$$

where $\sigma :\varepsilon =\sum_{ij}\sigma_{ij}\varepsilon_{ij}$ (Equation (A8)). Note that here **v**(**r**,*t*) represents the time derivative of the displacement vector, **v**(**r**,*t*) = ∂ **u**(**r**,*t*)/∂t, and not the flow velocity.

In homogeneous isotropic materials with zero flow velocity and hydrostatic pressure (σ_v_(**r**,*t*) = *p*(**r**,*t*) = 0), we derive the internal energy density, $e_{\mathrm{int}}\left(r,t\right)$, from Equation (75), using the Cauchy stress tensor, Equation (108), assuming that the Lamé parameters are homogeneous (∇*μ =* ∇*λ =* 0):

(115) $$\frac{\partial e_{\mathrm{int}}}{\partial t}+L_{q}\Delta T=2\mu \;\varepsilon \left(u\right):\varepsilon \left(v\right)+\lambda \;\left(\mathrm{div}\;u\right)\left(\mathrm{div}\;v\right)+s_{q}\text{,}$$

in component notation,

(116) $$\frac{\partial e_{\mathrm{int}}}{\partial t}+L_{q}\sum_{n}{\left(\partial_{n}\right)}^{2}T=2\mu \;\sum_{ij}\varepsilon_{ij}\left(u\right)\;\varepsilon_{ij}\left(v\right)+\lambda \sum_{kl}\left(\partial_{k}u_{k}\right)\left(\partial_{l}v_{l}\right)+s_{q}\text{,}$$

where Δ*T*(**r**,*t*) is the Laplace operator acting on a scalar field, *T*(**r**,*t*) (Equation (A5)). Assuming that the hydrostatic pressure is zero (*p*(**r**,*t*) = 0) and that there are no thermal effects (*L*_*q*_(**r**,*t*) = *s*_*q*_(**r**,*t*) = 0), we derive the internal energy equation for elasticity in homogeneous isotropic materials,

(117) $$\frac{\partial e_{\mathrm{int}}}{\partial t}=2\mu \;\varepsilon \left(u\right):\varepsilon \left(v\right)+\lambda \;\left(\mathrm{div}\;u\right)\left(\mathrm{div}\;v\right)\text{.}$$

### 6.4 Thermoelasticity

Stresses may also be generated in materials by changes in volume resulting from temperature variations. Thermoelasticity studies stresses caused by these thermal processes.

In linear thermoelasticity, we assume that the stresses are small, allowing a description of the total stress as the sum of mechanical stresses described by the Cauchy stress tensor of elasticity, **σ** (Equation (107)), and thermal stresses, **σ**_th_. Thus, the total stress, **σ**_tot_, can be expressed as

(118) $$\sigma_{\text{tot}}\left(r,t\right)=\sigma \left(r,t\right)-\sigma_{\text{th}}\left(r,t\right)\text{,}$$

where the thermal stress can be expressed as

(119) $$\sigma_{\text{t}\text{h}}=\beta \left(r,t\right)\;\Delta T\left(r,t\right)\text{,}$$

in component notation,

(120) $${\left(\sigma_{th}\right)}_{ij}=\beta_{ij}\;\Delta T\text{,}$$

where *β*_*ij*_ is a thermal expansion tensor and Δ*T*(**r**,*t*) = *T*(**r**,*t*) – *T*(**r**(*t*_0_),*t*_0_) is a small deviation in the temperature distribution relative to a reference distribution at *t* = *t*_0_. In the simplest case, *T*(r(*t*_0_),*t*_0_) is homogeneous (∇*T*(r(*t*_0_),*t*_0_) = 0) and independent of position, thus, in this case, Δ*T*(**r**,*t*) = *T*(**r**,*t*) – *T*_0_.

In isotropic materials, Equation (118) simplifies to (Equation (64) with **y**(**r**,*t*) = **u**(**r**,*t*))

(121) $$\sigma_{\text{t}\text{o}\text{t}}=2\mu \;\varepsilon \left(u\right)+\lambda \;\left(\mathrm{div}\;u\right)\;I_{3}-\beta \;\Delta T\left(r,t\right)\;I_{3}\text{,}$$

where *β* (**r**,*t*) is the *thermal expansion*, a scalar function.

The basic equations of linear thermoelasticity, including the momentum and energy equations, assuming homogeneous *μ*, *λ*, and *β* (∇ *μ =* ∇ *λ =* ∇ *β =* 0), can be obtained by incorporating the part of the stress tensor due to thermal conduction into Equations (113), (117). Thus, in linear materials under thermal stresses, we obtain the momentum equation,

(122) $$\rho_{0}\frac{\partial^{2}u}{\partial t^{2}}=\mu \;\Delta u+\left(\mu +\lambda \right)\nabla \left(\mathrm{div}\;u\right)+\beta \;\text{grad}\;\left(\Delta T\right)+f\text{,}$$

and the energy equation,

(123) $$\frac{\partial \varepsilon_{\mathrm{int}}}{\partial t}=2\mu \;\varepsilon \left(u\right):\varepsilon \left(v\right)+\lambda \left(\mathrm{div}\;u\right)\left(\mathrm{div}\;v\right)+\left(\beta \Delta T\right)\;\mathrm{div}\;v\text{.}$$

## 7 chemical reactions

We consider chemical reactions as described by physical chemistry the balance equation approach. Physical chemistry studies chemical interactions based on the principles of physics. The number of atoms is conserved under chemical reactions, since they do not create, destroy, or transfer atoms. As a consequence, the sum of numbers of each atom *k* in all reactants must be the same as those in all products,

(124) $$\sum_{i}{Z_{ki}\alpha }_{i}=\sum_{j}{Z_{kj}\alpha }_{j}\text{,}$$

where the sums are over all the reactants, *i*, and products, *j*, involved in the reaction, and *Z*_*ki*_ and *Z*_*kj*_ are numbers of atom *k* in the reactants and products. The $\alpha_{i}$-s and $\alpha_{j}$-s are referred to as the stoichiometric coefficients, determined only up to a multiplicative factor and typically reduced by dividing by their common denominator. The equation resonates with the underlying principle that chemical reaction equations must be stoichiometrically balanced.

As a chemical reaction proceeds, the law of mass conservation dictates that the total mass of all reactants and products remains constant:

(125) $$\sum_{i}M_{i}=\sum_{j}M_{j}\text{,}$$

where the sums are over all the reactants, *i*, and products, *j*, involved in the reaction. In general, the number of molecule *k* at any time can be calculated as *N*_*k*_ *= M*_*k*_*/m*_*k*_, where *m*_*k*_ is the molecular weight. Using this result, the mass conservation can be expressed as

(126) $$\sum_{i}N_{i}m_{i}=\sum_{j}N_{j}m_{j}\text{.}$$

### 7.1 Extensive and intensive quantities of chemical reactions

The extensive and intensive quantities for chemical reactions are the extent of reaction, *ξ*, and the chemical affinity, *A*. The extent of reaction describes the progress of the reaction over time. When no more reactant can participate in the reaction, the chemical affinity drops to zero, causing the reaction to stop.

During a chemical reaction, a small change in the energy of the system can be expressed as

(127) ΔE=TΔS–pΔV–AΔξ,

where the change in the entropy and volume are Δ *S = S*_2_ – *S*_1_ and Δ *V = V*_2_ – *V*_1_, and

(128) $$\Delta \xi =\xi_{2}\;–\xi_{1}=\sum_{j}\mu_{2j}\alpha_{2j}\;–\sum_{i}\mu_{1i}\alpha_{1i}\text{,}$$

where $\mu_{1i}$ and $\mu_{2j}$ are the chemical potentials at two consecutive time epochs, *t*_*1*_ and *t*_*2*_ (*t*_*1*_ → *t*_*2*_). At the end of the reaction, when the affinity drops to zero, *A* = 0, the total energy change will be

(129) ΔE=TΔS–pΔV,

as the chemical reaction changes the entropy and potentially the volume of the system.

### 7.2 Balance equation of mass with chemical reactions

If only physical processes take place, the mass of the different chemical components are conserved. However, in chemical reactions, only the total mass of the system is conserved, not those of the individual reactants. Consequently, the balance equation for the mass conservation of the individual reactants must contain net source terms (all sources minus sinks).

In chemical systems, the movements of chemical species are described by the time and position dependent mass density, $\rho_{k}\left(r,t\right)$, of a given reactant *k*. The convective flux density of reactant *k* is given by $\rho_{k}\left(r,t\right)v\left(r,t\right)$, assuming that the flow velocity, v(r,t), is the same for all reactants. The conductive flux density is originated from diffusion, and it can be expressed as $–D_{k}\left(r,t\right)\nabla \rho_{k}\left(r,t\right)$, where $D_{k}$ is the diffusion coefficient for reactant *k*, as derived from Fick's (first) law (Equation (166)). The balance equation describing the change in $\rho_{k}\left(r,t\right)$ can be derived from the mass conservation equation, Equation (55), with the source term generated by the chemical reaction, $\rho_{k}\left(r,t\right)\;r\left(r,t\right)$, which is determined by the rate of reaction, r(r,t), we obtain

(130) $$\frac{\partial \rho_{k}\left(r,t\right)}{\partial t}+\mathrm{div}\;\left[\;\rho_{k}\left(r,t\right)v\left(r,t\right)\;–D_{k}\left(r,t\right)\nabla \rho_{k}\left(r,t\right)\right]=\rho_{k}\left(r,t\right)\;r\left(r,t\right)\text{.}$$

If multiple reactions in the system change the quantity of reactant *k*, all of those source terms should be included on the right-hand side of the equation.

## 8 Electrodynamics

Classical electrodynamics is a branch of physics that studies electromagnetic interactions: interactions between electric charges and currents, electromagnetic waves, assuming that quantum mechanical effects are ignorable. We assume that the continuum mechanics approximation is valid and study classical electrodynamics (electrodynamics for short) based on the balance equation approach.

The balance equations for electric charge, electromagnetic momentum, and energy with the addition of the constitutive equations (Equations (131), (132)) give a complete description of electrodynamics. In this section, we determine these balance equations and derive from them the most famous and most frequently used equations of electrodynamics, Maxwell's equations.

We use the macroscopic description of electrodynamics in our derivations. In this approach of electrodynamics, the densities of the auxiliary fields, namely the dielectric polarization density, p(r,t), and magnetization density, m (**r**,*t*), are related to the electric and magnetic fields, **E**(**r**,*t*) and **H**(**r**,*t*), as

(131) p(r,t)=χ(r,t)E(r,t)

and

(132) $$m\left(r,t\right)=\chi_{m}\left(r,t\right)\;H\left(r,t\right)\text{,}$$

where **χ** and **χ**_*m*_ are the electric and magnetic susceptibilities of the material filling up the system. Note, that for linear homogeneous and isotropic materials **χ** and **χ**_*m*_ are scalars. However, for linear anisotropic materials, they are 2nd-rank tensors. The constitutive relations connect the electric and magnetic flux densities (also called electric displacement and magnetizing fields), **D**(**r**,*t*) and **B**(**r**,*t*), to the electric and magnetic fields, **E**(**r**,*t*) and **H**(**r**,*t*),

(133) $$D\left(r,t\right)=\epsilon_{e}\left(r,t\right)\;E\left(r,t\right)=\left(1+4\;\pi \;\chi \left(r,t\right)\right)\;E\left(r,t\right)\text{,}$$

and

(134) $$B\left(r,t\right)=\mu_{m}\left(r,t\right)\;H\left(r,t\right)=\left(1+4\;\pi \;\chi_{m}\;\left(r,t\right)\right)\;H\left(r,t\right)\text{,}$$

where the electric permittivity, $\epsilon_{e}$ (**r**,*t*), and the magnetic permeability, **μ**_*m*_(**r**,*t*), describe the microscopic properties of the material (e.g., Ref. [55]). Note, that for linear homogeneous and isotropic materials $\epsilon_{e}$ and **μ**_*m*_ are scalars, however, for linear anisotropic materials, they are 2nd-rank tensors. **D** is parallel to **E** and **B** is parallel to **H** only in isotropic materials.

### 8.1 Continuity equation: Charge conservation

In electrodynamics, the conservation of the relevant extensive quantity, namely the electric charge, is expressed by the charge continuity equation. From Equation (76), with *ρ*(**r**,*t*) identified with the electric charge density, *ρ*(**r**,*t*) = *ρ_e_*(**r**,*t*), and the (convective) flux density with the electric current density, $j_{e}\left(r,t\right)$,

(135) $${j\left(r,t\right)=j}_{e}\left(r,t\right)=\rho_{e}\left(r,t\right)\;v_{e}\left(r,t\right)\text{,}$$

where **v**_*e*_(**r**,*t*) is the velocity of the electric charges (Equation (16)), we derive the *charge continuity equation*,

(136) $$\frac{\partial }{\partial t}\;\rho_{e}\left(r,t\right)+\mathrm{div}\left[\;j_{e}\left(r,t\right)\right]=\frac{\partial }{\partial t}\;\rho_{e}\left(r,t\right)+\mathrm{div}\;\left[\rho_{e}\left(r,t\right)\;v_{e}\left(r,t\right)\right]=0\text{.}$$

The continuity equation for electric charge is often written identifying *ρ*(**r**,*t*) with the electric charge density. This notation could be misleading at first sight, as *ρ*(**r**,*t*) is usually associated with mass density. Note, that it is not the electric charge density, $\rho_{e}$, that is conserved, but rather the associated extensive quantity, the total electric charge, $Q=\int_{V}\rho_{e}\left(r,t\right)dV$, where *V* is a large enough volume to contain all the charges.

### 8.2 Balance equation for electromagnetic momentum

The electromagnetic momentum density is given by

(137) $$p_{\mathrm{EM}}\left(r,t\right)=\frac{1}{4\pi c}\;E\left(r,t\right)\times H\left(r,t\right)\text{.}$$

The electromagnetic momentum density is a vector field; thus, we must apply the balance equation for vectorial quantities, Equation (43), with the flux density tensor **J**(**r**,*t*) = **T**_EM_(**r**,*t*) specifying the electromagnetic momentum flux, we obtain

(138) $$\frac{1}{4\pi c}\;\frac{\partial }{\partial t}\left[E\times H\right]+\mathrm{Div}\;T_{\mathrm{EM}}=-\rho_{e}E-\frac{1}{c}\;j_{e}\times B\text{,}$$

where on the right-hand side are the electromagnetic source terms, with **j**_e_(**r**,*t*) representing the electric current density of free electrons. The electron current (flux) density in vacuum (or in a microscopic description) is given by Equation (135) (e.g., Ref. [56]).

In linear materials, the electron flux density, **j**_*e*_, is proportional to the gradient of the intensive quantity of the electrostatic process, namely the electric potential, *U*_*q*_(**r**,*t*), which generates it,

(139) $$j_{e}\left(r,t\right)=\sigma_{e}\left(r,t\right)\;\text{grad}\;U_{q}\left(r,t\right)=\sigma_{e}\left(r,t\right)\;E\left(r,t\right)\text{,}$$

where **E**(**r**,*t*) = grad *U*_q_(**r**,*t*) is the electric field and **σ**_*e*_ represents the electric conductivity (Equation (22) with **j**_e_(**r**,*t*) = **J**_cd_(**r**,*t*), y(**r**,*t*) = *U*_q_(**r**,*t*), and *L*_X_(**r**,*t*) = **σ**_*e*_). This is known as *Ohm's law* of electric conductivity. The electric conductivity, **σ**_*e*_, depends on material properties. In isotropic materials $\sigma_{e}\left(r,t\right)$ is a scalar function.

The electromagnetic momentum flux density can be expressed as

(140) $$T_{\text{EM}}=\frac{1}{4\pi }\;\left[\frac{1}{2}\;\left(E\cdot D+H\cdot B\right)\;I_{3}-\left(E⊗D+H⊗B\right)\right]\text{,}$$

where **I**_3_ is the 3D identity matrix. The flux density, **T**_EM_, can also be written as

(141) $$T_{\text{EM}}=-\frac{1}{4\pi }\;\left(E⊗D+H⊗B\right)+p_{EM}\;I_{3}\text{,}$$

where the electromagnetic pressure can be calculated as

(142) $$p_{EM}=\frac{1}{8\pi }\left[E\cdot D+H\cdot B\right]\text{.}$$

In electrodynamics, the Maxwell stress tensor, **T**_MX,_ is used instead of the electromagnetic momentum flux tensor (**T**_MX_ = –**T**_EM_).

There is an interesting analogy between the electromagnetic momentum flux density and the stress tensor of fluid dynamics. Equation (141) for **T**_EM_ can be written as

(143) $$T_{\mathrm{EM}}=\left[\frac{-1}{4\pi }\right]E⊗D+\left[\frac{-1}{4\pi }\right]H⊗B+p_{EM}\;I_{3}\text{.}$$

This equation can be obtained from the flux density tensor, Equation (86), by making the following substitutions: (1) substitute *ρ***v** and **v** with (−1/(4π*)***D** and **E** and substitute *ρ***v** and **v** with (−1/(4π*)***B** and **H**, in the first (convective) term of Equation (86); (2) substitute the effective pressure term, *p*_*eff*_ with the electrodynamic pressure, *p*_*EM*_ (Equation (142)), assuming that the deviatoric stress tensor is zero: **τ** = 0.

### 8.3 Balance equation for electromagnetic energy density: Maxwell's equations

We derive the macroscopic version of the basic equations of electrodynamics, known as Maxwell's equations, from the balance equation for electromagnetic energy density. In this version, the charges and currents are expressed at the macroscopic level. The total electric charges and total electric currents are the sum of free and bound charges, and the sum of free and bound currents. The dynamical Maxwell's equations can be derived from the differential version of the balance equation (Equation (42)), by using the electromagnetic energy density, *u*_*EM*_,

(144) $$u_{EM}=\frac{1}{8\pi }\;\left[E\cdot D+H\cdot B\right]\text{,}$$

as the density (*ρ*_*X*_(**r**, *t*) = *u*_*EM*_(**r**, *t*)), assuming that the electric permittivity and the magnetic permeability (ϵ_*e*_ (**r**, *t*) and **μ**_*m*_(**r**, *t*); Equations (133), (134)) are time-independent (e.g., Ref. [56]). Note that Maxwell's definition of electromagnetic energy density, as given in Equation (144), is problematic if ϵ_*e*_ and **μ**_*m*_ depend on time (e.g., Ref. [57]).

The flux density of the electromagnetic energy is given by the Poynting vector,

(145) $$j\left(r,t\right)=j_{\text{EM}}=\frac{c}{4\pi }\;E\times H\text{,}$$

and the source density is

(146) $$s_{X}\left(r,t\right)=s_{e}=-j_{e}\cdot E\text{,}$$

where the electric current density, **j**_e_(**r**,*t*)**,** of free electrons is given by Equation (139). By applying Equations (144), (145), (146), from the balance equation, given by Equation (42), we obtain the *balance equation for electromagnetic energy density*,

(147) $$\frac{1}{8\pi }\;\frac{\partial }{\partial t}\left[E\cdot D+H\cdot B\right]+\frac{c}{4\pi }\;\mathrm{div}\;\left[E\times H\right]=-j_{e}\cdot E\text{.}$$

Using the constitutive relations (Equations (133), (134)), from Equation (147), we obtain

(148) $$\frac{1}{8\pi }\;\frac{\partial }{\partial t}\left[\epsilon_{e}E^{2}+\mu_{m}H^{2}\right]+\frac{c}{4\pi }\;\mathrm{div}\;\left[E\times H\right]=-j_{e}\cdot E\text{.}$$

Multiplying Equation (148) by 4π/*c*, using the vector identity

(149) div[E×H]=H·[∇×E]−E·[∇×H],

and applying our assumption that ϵ _*e*_ and **μ**_*m*_ are time-independent, we obtain

(150) $$\left[\frac{1}{c}\frac{\partial D}{\partial t}-\nabla \times H+\frac{4\pi }{c}\;j_{e}\right]\cdot E+\left[\frac{1}{c}\frac{\partial B}{\partial t}+\nabla \times E\right]\cdot H=0\text{,}$$

where we used the constitutive equations (Equations (133), (134)) to bring back **D** and **H** thereby eliminating ϵ_*e*_ and **μ**_*m*_. The vectorial products of this equation with **E** and then with **H**, give the two dynamical Maxwell's equations, which describe how the electric and magnetic fields change over time,

(151) $$\nabla \times H=\frac{1}{c}\frac{\partial D}{\partial t}+\frac{4\pi }{c}\;j_{e}\text{,}$$

and

(152) $$\nabla \times E=-\frac{1}{c}\frac{\partial B}{\partial t}\text{,}$$

where **j**_e_ is given by Equation (139). Note that here we suppressed the dependence of the variables on position and time (**r** and *t*).

Taking the divergence of the two dynamical Maxwell's equations, ∇ · (Equation (151)) and ∇ · (Equation (152)), we obtain

(153) $$0=\frac{1}{c}\nabla \cdot \left(\frac{\partial D}{\partial t}\right)+\frac{4\pi }{c}\nabla \cdot j_{e}\text{,}$$

and

(154) $$0=-\frac{1}{c}\nabla \cdot \left(\frac{\partial B}{\partial t}\right)\text{,}$$

since the differential operator, div rot = (∇ · ∇×) applied to a vector field gives zero. By changing the order of ∇ and ∂/∂t operators in these equations (assuming that the fields are smooth) and using the continuity (equation (136)) in Equations (153), (154), we obtain

(155) $$\frac{\partial }{\partial t}\left[\nabla \cdot D-4\pi \rho_{e}\right]=0\text{,}$$

and

(156) $$\frac{\partial }{\partial t}\left[\nabla \cdot B\right]=0\text{.}$$

Integrating these equations and considering that, by definition, electric or magnetic fields cannot exist without corresponding electric or magnetic charges, we obtain the two static Maxwell's equations:

(157) $$\nabla \cdot D=4\pi \rho_{e}\text{,}$$

and

(158) ∇·B=0.

The crucial assumptions in our derivation of Maxwell's equations are that the electric permittivity and the magnetic permeability are time-independent. Note that the system of Maxwell's equations offers an amazingly compact description of the electromagnetic fields. From Maxwell's equations, we can derive the charge continuity equation (Equation (136)), the balance equation for electromagnetic momentum density (Equation (138)), and the balance equation for electromagnetic energy density (Equation (147)) from Maxwell's equations.

Maxwell's equations (Equations (151), (152), (157), (158)), combined with the constitutive relations (Equations (133), (134)), provide a closed system of equations that enables the derivation of the electromagnetic fields, **E**, **D**, **H**, and **B**. The system of Maxwell's equations is overdetermined: we have two vector equations (Equations (145), (146)) and two scalar equations (Equations (157), (158)) in 3D, which is equivalent to eight scalar equations for 6 variables (or two 3D vector quantities, namely **E** and **H**) by eliminating **D** and **B** using the constitutive equations, **D** = ϵ_*e*_**E** and **B** = **μ**_*m*_**H** (Equations (133), (134)). The charge distribution, *ρ*_*e*_(**r**,*t*), and the material constants, ϵ_*e*_(**r**), **μ**_*m*_(**r**), and *ρ*_*e*_(**r**,*t*), are assumed to be known. However, as mentioned earlier, by using charge conservation and the basic definition of fields (no charge no corresponding fields), we can derive Equations (157), (158) from Equations (151), (152), rendering Equations (157), (158) redundant.

Interestingly, although the dynamic and static Maxwell's equations are qualitatively different, it has been shown that all four equations are necessary for the integrability conditions to be satisfied for the system of equations [58]. Furthermore, it was also demonstrated that these integrability conditions prohibit the existence of magnetic monopoles [58].

### 8.4 Balance equation for internal energy from electromagnetic processes

Changes in the polarization and magnetization in materials cause changes in the internal energy density. The source term of the internal energy in electrodynamics arises from changes in polarization density, p(r,t), and magnetization density, m(r,t)**,**

(159) $$s_{q}\left(r,t\right)=E\cdot \frac{\partial p}{\partial t}+H\cdot \frac{\partial m}{\partial t}$$

In most applications the flow velocity of the material, **v**(**r**,*t*), is zero. Thus, we obtain the balance equation for the internal energy density originating from electromagnetic processes from Equation (75) with the source term given by Equation (159), and applying **v**(**r**,*t*) = **q**(**r**,*t*) = 0,

(160) $$\frac{\partial e_{\mathrm{int}}}{\partial t}=E\cdot \frac{\partial p}{\partial t}+H\cdot \frac{\partial m}{\partial t}\text{.}$$

## 9 Applications of balance equations

### 9.1 Diffusion: Fick's equation

Diffusion is a process of mass transfer in time. Diffusion causes the distribution of chemical species to become more uniform in space. In the context of simple diffusion, where only chemical processes are important, the chemical potential, *μ*(**r**,*t*), is the sole relevant inhomogeneous intensive quantity. In this case, diffusion is driven by inhomogeneities in the chemical potential causing fluxes in its associated extensive quantity, namely the number of species in system (*N*), expressed by their number density, *n*(**r**,*t*) (Table 2). In applications of diffusion, the mass density, *ρ*(**r**,*t*), is often used instead of the number density, and the flux of number of species becomes mass flux. The relevant balance equation in this context the mass conservation equation. Since the relevant quantities are scalars, the source density is also a scalar, while the flux density is a vector quantity.

In this case, furthermore, it is assumed that the velocity of the matter is zero, **v**(**r**,*t*) = 0, thus the convective flux density is zero **j**_cv_(**r**,*t*) = 0, and that there are no source terms, *s*_X_(**r**,*t*) = 0. The conductive flux, **j**_cd_(**r**,*t*), is generated by the inhomogeneities in the relevant intensive quantity, the chemical potential, *μ* (**r**,*t*). Assuming a linear material, **j**_cd_(**r**,*t*) can be expressed as

(161) $$j_{\text{cd}}\left(r,t\right)=L_{D}\left(r,t\right)\nabla \mu \left(r,t\right)\text{,}$$

where *L*_*D*_(**r**,*t*) is the conduction coefficient (Equation (22) with *y*(**r**,*t*) = *μ*(**r**,*t*) and *L*_*X*_(**r**,*t*) = *L*_*D*_(**r**,*t*)). Under these assumptions, the generic balance equation for scalar extensive quantities, Equation (42), contains the mass density, *ρ*_*X*_(**r**,*t*) = *ρ*(**r**,*t*), the mass flux density by conduction, **j**(**r**,*t*) = **j**_cd_(**r**,*t*) (Equation (161)), and no source terms, *s*_*X*_(**r**,*t*) = 0, and thus we obtain the balance equation for mass density,

(162) $$\frac{\partial \rho }{\partial t}+\mathrm{div}\left[L_{D}\nabla \mu \right]=0\text{.}$$

This is essentially the diffusion equation, where the conduction coefficient, *L*_*D*_(**r**,*t*), has the role of the diffusion coefficient. We can transfer Equation (162) into a well-known form using the relations between thermodynamic variables: the dependence of the chemical potential on the mass density and the temperature, *μ* = *f* (*ρ*, *T*). Thus, the gradient of *μ* (**r**,*t*) can be expressed as

(163) $$\nabla \mu ={\left(\frac{\partial \mu }{\partial \rho }\right)}_{T}\nabla \rho +{\left(\frac{\partial \mu }{\partial T}\right)}_{\rho }\nabla T\text{,}$$

and, since we assume that ∇*T*(**r**,*t*) = 0, we obtain

(164) $$L_{D}\nabla \mu =L_{D}{\left(\frac{\partial \mu }{\partial \rho }\right)}_{T}\nabla \rho \text{.}$$

In physics and engineering, the diffusion constant, *D*(**r**,*t*), is used

(165) $$D=L_{D}\nabla \mu =-L{\left(\frac{\partial \mu }{\partial \rho }\right)}_{T}\text{,}$$

which is *Fick's (first) law*, and we obtain,

(166) $$\frac{\partial \rho }{\partial t}-\mathrm{div}\;\left[D\nabla \rho \right]=0\text{.}$$

If the diffusion constant, *D*, is homogeneous, we obtain *Fick's equation*, or *Fick's second law*,

(167) $$\frac{\partial \rho }{\partial t}-D\Delta \rho =0\text{,}$$

where Δ *ρ*(**r**,*t*) is the Laplace operator acting on a scalar field, *ρ*(**r**,*t*) (Equation (A5)).

### 9.2 Thermal conduction: Fourier's equation

Thermal Conduction is the process of heat being transferred from the hotter to the cooler part of a material. We assume that only thermal processes are acting in simple heat conduction. In this case, the only relevant inhomogeneous intensive quantity is the temperature. Temperature inhomogeneities cause changes in the associated extensive quantities of thermodynamic processes, namely the entropy, *S* (Table 2). The generated entropy flux results in an energy flux, which is also known as the heat flux. The relevant balance equation that describes the simple thermal conduction is the balance equation for internal energy. Thus, to derive Fourier's equation, we use the internal energy conservation equation. The relevant intensive quantity, temperature, *T*(**r**,*t*), is a scalar function, resulting in a scalar associated source density, while the generated flux density is a vector quantity.

In the simplified model of thermal conduction, it is assumed that no source terms exist, *s*(**r**,*t*) = 0, and that the flow velocity of matter is zero, **v**(**r**,*t*) = 0. As a consequence, the convective flux density is zero, **j**_cv_ = 0, and the gradient of the relevant intensive quantity, namely the temperature, generates the heat flux density, **q**(**r**,*t*), by conduction,

(168) $${q\left(r,t\right)=j}_{\text{cd}}\left(r,t\right)=L_{T}\left(r,t\right)\nabla T\left(r,t\right)\text{,}$$

where *L*_*T*_ (**r**,*t*) is the conduction coefficient (Equation (22) with y(**r**,*t*) = *T*(**r**,*t*) and *L*_*X*_ (**r**,*t*) = *L*_*T*_ (**r**,*t*)). In physics and engineering, heat conduction, or thermal conductivity, *λ*, is used, *λ*(**r**,*t*) = − *L*_*T*_ (**r**,*t*), thus we obtain

(169) q(r,t)=–λ(r,t)∇T(r,t).

With these assumptions, the balance equation for the internal energy density, Equation (42), with *ρ*_*X*_(**r**,*t*) = *e*_*int*_(**r**,*t*), *s*_*X*_(**r**,*t*) = 0, and **j**(**r**,*t*) = **q**(**r**,*t*) (Equation (169)) becomes

(170) $$\frac{\partial e_{\mathrm{int}}}{\partial t}–\mathrm{div}\left[\lambda \nabla T\right]=0\text{.}$$

The dependence of the internal energy density on the density and the temperature is given by the equation of state, *e*_*int*_ = *f* (*ρ*,*T*). We expand the derivative of *e*_*int*_ (**r**,*t*) as

(171) $$\frac{\partial e_{\mathrm{int}}}{\partial t}=\frac{\partial e_{\mathrm{int}}}{\partial \rho }\frac{\partial \rho }{\partial t}+\frac{\partial e_{\mathrm{int}}}{\partial T}\frac{\partial T}{\partial t}\text{.}$$

If the density is stationary, ∂*ρ*/∂t = 0, the first term is zero, and thus

(172) $$\frac{\partial e_{\mathrm{int}}}{\partial t}=c_{v}\rho \frac{\partial T}{\partial t}\text{,}$$

where *c*_v_ is the specific heat at constant volume (∂*e*_*int*_/∂*T* = *c*_v_ *ρ*). Assuming that the thermal conductivity is homogeneous, from the balance equation, Equation (170), we obtain,

(173) $$c_{v}\rho \frac{\partial T}{\partial t}=\lambda \;\Delta T\text{,}$$

where Δ*T*(**r**,*t*) is the Laplace operator acting on a scalar field, *T*(**r**,*t*) (Equation (A5)). Dividing both sides of Equation (173) by *c*_v_ (assuming that it is not zero), we obtain Fourier's equation,

(174) $$\frac{\partial T}{\partial t}=a\;\Delta T\text{,}$$

where *a* = *λ*/(*c*_v_ *ρ*) is the thermal diffusivity.

### 9.3 Non-viscous Newtonian fluids: Euler equation

In non-viscous Newtonian fluids the viscosity coefficients are assumed to vanish (*μ* (**r**,*t*) = *ζ* (**r**,*t*) = 0), thus the Cauchy tensor becomes $\sigma_{p}\left(r,t\right)=-p\left(r,t\right)\;I_{3}$ (Equation (60)). With this assumption, we obtain the Euler equation, the most frequently used and well-known momentum balance equation of hydrodynamics, from the Navier–Stokes equation (Equation (91)),

(175) $$\rho \left[\frac{\partial v}{\partial t}+\left(v\cdot \nabla \right)v\right]=-\nabla p+f\text{.}$$

Note that this is essentially Newton's second law expressed in densities of the extensive physical quantities: the acceleration of a cell in a fluid with density *ρ*(**r**,*t*) depends on the sum of the forces, *f* (**r**,*t*) – ∇*p*(**r**,*t*), acting on it.

### 9.4 Bernoulli's equation

Bernoulli's equation is the most renowned equation in fluid mechanics. It can be straightforwardly derived from the Euler equation (Equation (175)) by assuming that the external force density is provided only by gravity, **f**(**r**,*t*) = –*ρ*(**r**,*t*) **g**(**r**,*t*), where **g**(**r**,*t*) is the gravitational acceleration. From Equation (175) we obtain

(176) $$\rho \frac{\partial v}{\partial t}+\rho \left(v\cdot \nabla \right)v=-\rho g-\nabla p\text{.}$$

Assuming a stationary flow (∂**v**/∂*t* = 0) and integrating Equation (176) along a streamline yields:

(177) $$\frac{1}{2}v^{2}+\frac{p}{\rho }+gz=constant\text{,}$$

where we assume that the *z* axis is parallel to the direction of the gravitational field. This is *Bernoulli's equation*.

### 9.5 Wave equation, Helmholtz equation

Consider small perturbations in an inviscid fluid (*μ*(**r**,*t*) = *ζ*(**r**,*t*) = 0) in rest (v(r,t) = 0). We write the density, pressure, and velocity of the fluid as $\rho \left(r,t\right)=\rho_{0}+\epsilon \bar{\rho }\left(r,t\right)$, $p\left(r,t\right)=p_{0}+\epsilon \bar{p}\left(r,t\right)$, $u\left(r,t\right)=\epsilon \bar{u}\left(r,t\right)$, where ϵ is a small perturbation, $\bar{\rho }\left(r,t\right)$, $\bar{p}\left(r,t\right)$, and $\bar{u}\left(r,t\right)$ are the perturbed density, pressure and velocity fields of the fluid. For these perturbations, from Equation (76), we obtain the linearized continuity equation,

(178) $$\frac{\partial }{\partial t}\bar{\rho }\left(r,t\right)+\rho_{0}\nabla \cdot \left[\bar{v}\left(r,t\right)\right]=0\text{,}$$

and the linearized Navier—Stokes equations (Equation (91)),

(179) $$\rho_{0}\frac{\partial \bar{v}}{\partial t}+\nabla \bar{p}=0\text{,}$$

where we dropped all terms higher order than one in ϵ. The perturbed pressure and density are related as

(180) $$\bar{p}\left(r,t\right)=\frac{\partial \bar{p}}{\partial \bar{\rho }}\bar{\rho }\left(r,t\right)=c_{s}^{2}\;\bar{\rho }\left(r,t\right)\text{,}$$

where $c_{s}$ is the sound speed. With Equation (180), the perturbed linearized Navier—Stokes equations becomes

(181) $$\rho_{0}\frac{\partial \bar{v}}{\partial t}+c_{s}^{2}\nabla \bar{\rho }=0\text{.}$$

Taking the gradient of the continuity equation and the divergence of the momentum equation, using the continuity and momentum and equations to introduce the time dependent terms, and assuming that $\nabla \times \bar{v}=0$, we obtain the wave equations,

(182) $$\frac{\partial^{2}\bar{\rho }}{\partial t^{2}}-c_{s}^{2}\;\Delta \bar{\rho }=0\text{,}$$

and

(183) $$\frac{\partial^{2}\bar{v}}{\partial t^{2}}-c_{s}^{2}\;\Delta \bar{v}=0\text{,}$$

where Δ is the Laplace operator, Δ=∇·∇ (see Appendix A).

The Helmholtz equation represents a time-independent form of the wave equations. For a generic scalar function, u(r,t) (e.g., density in Equation (182) or the *x* component of the velocity field of Equation (183)), assuming that the spatial and temporal dependence of the solution is separable, u(r,t)=A(r)B(t), we can write Equation (182) (with $\bar{\rho }\left(r,t\right)=u\left(r,t)\right)$, as

(184) $$\frac{\Delta A}{A}\left(r\right)=\frac{1}{c_{s}^{2}\;B\left(t\right)}\;\frac{\partial^{2}B}{\partial t^{2}}\left(t\right)\text{.}$$

Since the left-hand side depends only on the special variable (r) and the right-hand side only on time (t), they both should be equal to a constant. This constant is usually written as $-k^{2}$, for convenience, thus we obtain the wave equations,

(185) $$\frac{\Delta A}{A}\left(r\right)=-k^{2}\text{,}$$

and

(186) $$\frac{1}{c_{s}^{2}\;B\left(t\right)}\;\frac{\partial^{2}B\left(t\right)}{\partial t^{2}}=-k^{2}\text{.}$$

The first is a time independent wave equation, it is called the Helmholtz equation,

(187) $$\Delta A\left(r\right)=-k^{2}\;A\left(r\right)\text{.}$$

From a mathematical point of view, the Helmholtz equation is the eigenvalue problem for the Laplace operator (Δ).

### 9.6 The fundamental equation of hydraulics

In engineering, hydraulics studies the mechanical properties and usage of liquids (e.g., water or oil). In hydraulics applications, the fluid can often be assumed incompressible. The flow is driven by the change in the relevant intensive quantity, the pressure gradient. The pressure gradient generates momentum flux described by the Navier—Stokes equations. It is often assumed that the fluid has uniform constant density (*ρ*(**r**,*t*) = *ρ*_0_) and kinematic viscosity (*ν*(**r**,*t*) = *ν*), and moves under a conservative force (e.g., gravity). In this case, the incompressible Navier–Stokes equation (Equation (94)), considering a conservative external force that generates acceleration, **a**(**r**,*t*) = – ∇ φ (**r**,*t*), and assuming a uniform density, *ρ*(**r**,*t*) = *ρ*_0_, we obtain the momentum balance equation,

(188) $$\frac{\partial v}{\partial t}+\left(v\cdot \nabla \right)v-\nu \Delta v=-\nabla h\text{,}$$

where, *ν* is the kinematic viscosity, and as customary, we defined the hydraulic head, $h=p/\rho_{0}+\varphi$. Equation (188) is the *fundamental equation of hydraulics*.

### 9.7 Pressure Poisson equation

Pressure waves in incompressible fluids can be described by the pressure Poisson equation. The balance equation for momentum for incompressible fluids (*ρ*(**r**,*t*) = const.) can be written as

(189) $$\frac{\partial v}{\partial t}+\left(v\cdot \nabla \right)\;v=\nu \;\Delta v-\frac{1}{\rho }\nabla p\text{,}$$

where ν is the kinematic viscosity (Equation (94)). Taking the divergence of both sides, and switching the order of differential operator (assuming smooth enough functions), and using the incompressible constraints (divv=0), we obtain

(190) Δp=−ρ(v·∇)v.

This is called the pressure Poisson equation.

### 9.8 Fluid flow through porous media

Fluid flow through porous media is a branch of fluid mechanics, that studies how fluids flow through a porous medium, such as water through send, of other porous material. One of the most important properties of porous media is the permeability of the media, which describes the resistance of the material against the flow. It is usually assumed that the velocity of the flow is linear in the microscopic velocity of the fluid. This relationship is parameterized by a coefficient called the porosity of the material. Fluid flow through porous media has applications in many branches of engineering and science (e.g., chemical, aeronautical, and reservoir engineering).

Fluid flow through a porous media is driven by the change in the relevant intensive quantity, the pressure gradient. The pressure gradient generates momentum flux described by the Navier—Stokes equations. In applications, it is It is usually assumed that the medium is homogenous, isotropic. The Navier—Stokes equations (Equation (91)), with incompressible fluid, divv=0, and a stationary, slow laminar flow in isotropic porous material becomes

(191) 0=−∇p(r,t)+f(v),

where ∇p(r,t), the hydraulic gradient, and f(v), is the viscous resisting force density, a function of the microscopic velocity, v(r,t). Assuming that the viscous resisting force is linear with the flow velocity, u(r,t), it can be expressed as

(192) $$f\left(v\right)=-\frac{\mu }{k}u\left(v\right)\text{,}$$

where μ is the dynamical viscosity and *k* is the permeability of the medium. Assuming that the velocity of the flow is linear in the microscopic velocity, u(r,t)=εv(r,t), where ε the is the porosity of the material, from Equations (191), (192), we obtain

(193) $$v\left(r,t\right)=-\frac{k}{\varepsilon \mu }\nabla p\left(r,t\right)\text{.}$$

From this equation, we obtain *Darcy's law*: the volumetric flux, the rate of volume flow across unit area, **q**(**r**,*t*) (e.g., in m^3^/sec/m^2^ = m/sec),

(194) $$q\left(r,t\right)=-\frac{k}{\mu }\nabla p\left(r,t\right)\text{,}$$

Where the permeability of the material, *k* (r,t), is a scalar function (note that in anisotropic materials *k* is a tensor) and ∇p(r,t) is also called the hydraulic gradient. Darcy's law states that in linear materials, the volumetric flux is proportional to the pressure gradient and inversely proportional to the dynamical viscosity, μ(r,t).

A special case of Darcy's law is the *Carman–Kozeny equation*, used to calculate the pressure drop of a fluid flow through a packed bed of solids. In chemical engineering, a packed bed is a hollow tube (or pipe, etc.) filled with a packing material to improve contact between two phases of material inside. In this case, the superficial velocity, the effective velocity of one phase of interest, $u_{s}\left(r,t\right)$, of the fluid, can be expressed as

(195) $$u_{s}\left(r,t\right)=-\frac{\kappa }{\mu }\nabla p\left(r,t\right)\text{,}$$

where κ is the single-phase permeability,

(196) $$\kappa =\Phi_{s}^{2}\frac{\varepsilon^{3}d_{P}^{2}}{180{\left(1-\varepsilon \right)}^{2}}\text{,}$$

where $\Phi_{s}$ is the sphericity of the particles in the packed bed ($\Phi_{s}=1$ for spherical particles), $d_{P}$ is the diameter of a spherical particle with the same volume as the actual particles, and ε is the porosity of the material.

### 9.9 Reacting fluid flows

Reacting fluid flows apply fluid dynamic models to systems undergoing chemical reactions with many applications. Reacting flows must satisfy the conservation of mass, momentum, and energy taking into account the effect of chemical reactions.

The continuity equation leads to the equation of mass conservation for each species, *i*. Assuming *N* different species (*i* = 1, 2, …or *N*), and expressing their continuity equations in terms of $Y_{i}\left(r,t\right)$, the mass fraction fractions of species *i*, $Y_{i}=m_{i}/m$ (where $m_{i}$ and m are the mass of species *i* and the total mass), from Equation (130) we derive

(197) $$\frac{\partial \rho Y_{i}}{\partial t}+\nabla \cdot \left[\;\rho Y_{i}\;v+\nabla \cdot j_{i}\;\right]=r_{i}\text{,}$$

where **v** (r,t) is the flow (bulk) velocity, $j_{i}\left(r,t\right)$ is the flux density due to diffusion and $r_{i}\left(r,t\right)$ is the production rate due to chemical reactions of species *i*. The total mass density, ρ(r,t), satisfies the mass continuity equation, Equation (130).

The conservation of momentum in fluid dynamics requires that the Navier–Stokes equations be satisfied. Assuming that the flow carries all the species at velocity v(r,t), from Equation (57), we obtain

(198) $$\frac{\partial \rho v}{\partial t}+\mathrm{Div}\left[\rho \;v⊗v\;–\;\sigma \right]=\rho \sum_{i}Y_{i}\;f_{i}\text{,}$$

where σ(r,t) is the Cauchy stress tensor, Equation (82), $f_{i}$ is the body force acting on species *i*, and the summation runs over all species (*i* = 1, 2, …*N*).

When considering the conservation of the total energy of the fluid, the balance equation for the energy density, e(r,t), must include the contributions from each species. From Equation (95), we obtain

(199) $$\frac{\partial e}{\partial t}+\nabla \cdot \left[ev\;–\;\sigma \;v+q\right]=\rho \sum_{i}\left(\;Y_{i}\;f_{i}\right)\cdot v+\sum_{i}\left(f_{i}\cdot \;j_{i}\right)\text{,}$$

where q(r,t) is the heat flux density vector (Equation (169)), $j_{i}$ is the flux density vector of diffusion of species *i*, and we used $s_{q}\left(r,t\right)=\rho \sum_{i}\left(\;Y_{i}\;f_{i}\right)\cdot v+\sum_{i}\left(f_{i}\cdot \;j_{i}\right)$ for the source term (*i* = 1, 2, …*N*). Mass conservation demands that $\sum_{i}r_{i}=\sum_{i}\;j_{i}=0$ and $\sum_{i}Y_{i}=1$.

### 9.10 Traffic flows: the Lighthill–Whitham–Richards equation

The balance equation method proves highly useful in fields beyond physics. We demonstrate the effectiveness of this method with a well-known problem: optimizing vehicle traffic flows of vehicles. The first large-scale treatment of traffic flows was developed by Lighthill amd Whitham [59], followed shortly by the introduction of shock waves [60]. From the continuity equation, we derive the basic equation of traffic flows, the Lighthill–Whitham–Richards equation. Our analysis assumes a single road without junctions. We identify the density, ρ(x,t), with the vehicle density (ρ=N/Δx, where N is the number of vehicles within Δx, x is the measured length along the road), and the flux density with the flux density of vehicles, j (x,t) = ρ(x,t)V(x,t), where V(x,t) is the average velocity of the vehicles at point *x* at time *t*. With these assumptions, from the continuity equation, Equation (76), in one dimension, we obtain

(200) $$\frac{\partial \rho \left(x,t\right)}{\partial t}+\frac{\partial }{\partial x}\left[\rho \left(x,t\right)V\left(x,t\right)\right]=0\text{.}$$

This equation describes how the vehicle density changes over time, assuming they move with velocity, V(x,t).

Equation (200) has two unknown functions, ρ(x,t) and V(x,t). Lighthill, Whitham, and Richards [59,60] closed the equation using the approximation that the velocity at *x* and time *t* depends the instantaneous density at the same time: V(x,t)=V(ρ(x,t)). They suggested to determine the form of the V(ρ(x,t)) function from fitting to data. Using this approximation in Equation (200), and the chain rule, we obtain the *Lighthill–Whitham–Richards equation* of traffic flows,

(201) $$\frac{\partial \rho \left(x,t\right)}{\partial t}+\frac{d\left(\rho V\left(\rho \right)\right)}{d\rho }\frac{\partial \rho \left(x,t\right)}{\partial x}=0\text{,}$$

where (ρV(ρ)) is considered to be a function of ρ.

## 10 Physics modules for physics-informed ML

### 10.1 The algorithm

The balance equations can be used to design physics modules based on a unified algorithm that can then be incorporated into PIML methods to solve complex systems. The physics modules can be developed by following these steps:

1. Identify all inhomogeneous intensive quantities driving the changes in the system, along with their associated extensive quantities.
2. Determine all sources and sinks as well as the fluxes of the associated extensive quantities.
3. Formulate the relevant balance equations.
4. Establish relations between the relevant quantities to close the equations.
5. Apply the derived system of equations within the machine learning module including the initial and boundary conditions to solve the complex problem.

These steps provide a framework for developing algorithms for PIML software.

Note that in many applications, we may assume that the fluxes of extensive quantities are proportional to the first derivatives of the relevant inhomogeneous intensive quantities (see, for example, Table 4).

### 10.2 Example: Physics module for Eulerian hydrodynamics

To illustrate our proposed method for developing a physics module using balance equations to integrate physics into machine learning solutions for complex systems, we apply it to an Eulerian hydrodynamical system. An Eulerian fluid is a special case of a Newtonian fluid, it is characterized by zero viscosity, no thermal processes, and no diffusion.

1) Identify all possible inhomogeneous intensive quantities driving the changes in the system and their associated extensive quantities.

In hydrodynamics we may encounter mechanical, thermal, and chemical processes. The assumption of zero viscosity implies that viscous coefficients are zero (*μ* = 0 and *ζ* = 0); the absence of thermal processes indicates that neither heat fluxes nor heat sources are present; no diffusion suggests that the chemical potential is homogeneous, ▽*μ*_*1*_ = 0 (assuming only one species is present). Thus, apart from the convective fluxes of the relevant extensive quantities, only pressure inhomogeneities drive changes in the system.

Thus, only inhomogeneities in the pressure drive changes in the system apart from the convective fluxes of the relevant extensive quantities.

2) Determine all sources and sinks, as well as fluxes of the associated extensive quantities.

We assumed that the external force density is the only source of momentum and energy, with no thermal heating present. Therefore, there is no heat flux. The relevant extensive quantities are mass, momentum, and total energy. The densities of these relevant quantities, ρ(r,t), p(r,t), and *e*(**r**,*t*), and their sources and fluxes are summarized in Table 5. Note that in the momentum fluxes ρv⊗v and –p **I**_**3**_ are 3-dimensional 2nd rank tensors, and **I**_**3**_ is the 3D identity tensor.

3) Formulate the balance equations for the densities of the relevant extensive quantities.

**Table 5:** Relevant extensive quantities of Eulerian hydrodynamics and their sources and fluxes.

Relevant extensive quantity	Source	Flux	
		Convective	Conductive
Mass [ρ(r,t)]	0	*ρ*(r,t)v(r,t)	0
Momentum [p(r,t)]	f(r,t)·v(r,t)	ρ(r,t)v(r,t)⊗v(r,t)	–p(r,t)**I**_**3**_
Total energy [e(r,t)]	e(r,t)·v(r,t)	e(r,t)v(r,t)	–p(r,t)v(r,t)

We need the balance equations for ρ(r,t), p(r,t), and *e*(**r**,*t*), which correspond to the equations of mass, momentum and energy conservation.

Under our assumptions, the mass balance equation simplifies to the mass continuity equation (Equation (76)),

(202) $$\frac{\partial }{\partial t}\;\rho \left(r,t\right)+\mathrm{div}\left[\rho \left(r,t\right)\;v\left(r,t\right)\right]=0\text{;}$$

the momentum balance equation can be expressed as (Equation (89) with *μ* = 0 and *ζ* = 0),

(203) $$\frac{\partial \rho v}{\partial t}+\mathrm{Div}\left[\rho \;v⊗v\right]+\nabla p=f\text{,}$$

where we used Div(–**σ)** = Div(pδ)=∇p; or using the convective form, we obtain the Euler equation in its usual form (Equation (175)),

(204) $$\rho \left[\frac{\partial v}{\partial t}+\left(v\cdot \nabla \right)v\right]+\nabla p=f\text{;}$$

and the balance equation for the total energy density (Equation (99) with *μ* = *ζ* = $L_{q}=s_{q}=0$) becomes

(205) $$\frac{\partial e}{\partial t}+\mathrm{div}\;\left(e\;v\right)+v\cdot \nabla p+p\;\left(\mathrm{div}\;v\right)=f\cdot v\text{.}$$

4) Establish relations between the relevant quantities to close the equations.

Up till now, we have 5 equations with 6 unknown scalar functions (three scalar and one vector variables: ρ(r,t), *e*(**r**,*t*), *p*(**r**,*t*), and v(r,t)). This system of equations can be closed using the constitutive relation for the ideal gas, known as the equation of state. Assuming calorically perfect gas, the equation of state can be expressed as *p*(**r**,*t*) = (γ(r,t)–1) *e*_*int*_(**r**,*t*), where *e*_*int*_(**r**,*t*) is the internal energy density and γ (**r**,*t*) is the adiabatic index. With this equation of state, the energy density, *e* = (1/2)*ρ*v^2^ + *e*_*int*_, can be expressed as

(206) $$e=\frac{1}{2}\rho v^{2}+\frac{p}{\gamma -1}\text{.}$$

The balance equations for mass and momentum along with the ideal gas law linking pressure and energy (Equations (202), (204), (205), (206)), yield a closed system of equations: 6 equations with 6 unknown scalar functions, assuming that **f**(**r**,*t*) and γ (**r**,*t*) are known.

5) Apply the derived system of equations within the machine learning module including the initial and boundary conditions to solve the complex problem.

The constraints can be incorporated in the ML procedure by introducing a loss function, for example. The general approach considers a differential equation representing a physical process, u(t,x), e.g., a conservation law, for which the model is being trained

(207) $$u_{t}+N\left[u\right]=0;x\in \Omega ;t\in \left[0,T\right]$$

where N[·] is a nonlinear differential operator, Ω is a subset of $R^{n}$, and t is time (path parameter). A method of incorporating physical constraints into an ML context using the collocation method for the numerical solution of differential equations was developed by Raissi et al. [2]. The collocation method determines the approximate solution of a PDE, by requiring the equation to be satisfied at fixed specific points. These points are referred to as collocation points. Following Raissi et al., we introduce a function

(208) $$f\left(t,x\right)\equiv u_{t}+N\left[u\right]$$

defined over collocation points. The loss function for the original problem is defined as

(209) $${MSE}_{u}=\frac{1}{N_{u}}\sum_{i=1}^{N_{u}}{\left|u\left(t_{u}^{i},x_{u}^{i}\right)-u^{i}\right|}^{2}$$

where $\left\{\left(t_{u}^{i},x_{u}^{i}\right)|i=1,\ldots ,N_{u}\right\}$ represent initial and boundary condition training data for u(t,x). Then we introduce a loss function for f,

(210) $${MSE}_{f}=\frac{1}{N_{f}}\sum_{i=1}^{f}{\left|f\left(t_{f}^{i},x_{f}^{i}\right)\right|}^{2}\text{,}$$

computed at the collocation points: $\left\{\left(t_{f}^{i},x_{f}^{i}\right)|i=1,\ldots ,N_{f}\right\}$. The original problem of minimizing ${MSE}_{u}$ is then replaced by the problem of minimizing: ${MSE}_{u}+{MSE}_{f}$.

## 11 Examples of balance equation method for PIML

In this section, we provide some simple worked out examples of the use of our method, starting from the balance equations to the implementation of NN based on DeepXDE.

The balance equation framework enables physics constraints to be include in PIML by specifying the relevant balance equations and the corresponding constitutive equations. These can be combined and linked to PIML as terms in the loss function. These terms can be weighted. The relevant domain (geometry) must be specified along with the relevant initial and boundary conditions. The neural network architecture is then specified and trained. In the case of inverse problems, it is necessary to parameterize the PDE and communicate these parameters to the neural network for updating during the training process.

Lu et al. [61] offer the following algorithm for forward problems of PIML, which we have generalized to include the inverse problems:

1. Construct an NN: f(x;θ,λ), where θ are nodal parameters and λ are PDE parameters.
2. Specify two data sets for training: $T_{f}$ for the PDE and $T_{c}$ for the constraints.
3. Specify the loss function as the weighted sum of PDE and constraint losses: $L\left(\theta ;T\right)=w_{f}L_{f}\left(\theta ,\lambda ;T_{f}\right)+w_{c}L_{f}\left(\theta ,\lambda ;T_{c}\right)\;;\;w_{f},w_{c}\;are\;the\;weights$.
4. Train the NN to find : $\theta^{*},\lambda^{*}=\underset{\theta ,\lambda }{\mathrm{argmin}}L\left(\theta ;T\right)$.

For the forward problem, λ are fixed and can be omitted. In the case of inverse problems, it is necessary to parameterize the PDE and communicate these parameters to the neural net for updating during the training process.

There are several examples of PINN software libraries with open licenses that can be found on the Internet. Our goal is to build a bridge between a wide range of classical physics domains and one of these already well-developed packages. Accordingly, we have chosen a PINN implementation that seems to be particularly flexible and accessible, especially for new users of PINN technology: DeepXDE (https://pypi.org/project/DeepXDE/). Although for specific problems one may need a very specific NN design, DeepXDE itself can be adapted in some cases, as illustrated by Jha and Mallik [62].

We now consider examples of two basic forms of PDEs, the hyperbolic (Burger's equation) and the elliptic PDE (Hagen-Poiseuille and Poisson's equations).

Data must be supplied to an NN as a linear array, more precisely a data structure defined in in the TensorFlow and PyTorch packages as a “tensor.” Time t is represented as a single element along with x, which itself can represent multiple coordinates. To construct an input tensor one must stack the input elements, i.e., the tensor: [[t,x,y,z]].

Tensors as data structures should not be confused with tensors in physics, where coordinate transformations are understood to transform in a specific, physically meaningful way. Tensors in TensorFlow or PyTorch are a generalization of the array data structure adapted for use with GPUs and to support auto-differentiation, i.e., tensors are arrays that carry metadata. Syntactically, tensors are distinguished from arrays by the use of double parentheses, e.g., the array [[1], [2], [3], [4]]: ∼ the tensor: [[1], [2], [3], [4]].

### 11.1 Burger's equation

Our first example is based on the viscous Burger's equation. The inviscid Burger's equation is a prototype for conservation equations that can develop discontinuities (shock waves). We use this example to show how to solve time dependent PDE with DeepXDE, and how to use different optimizers for more effective training. We also show how to approach systems with weak solutions, in the case of Burger's equation, the shock wave solutions of the inviscid form. We can approximate these weak solutions by setting the kinematic viscosity very small.

#### 11.1.1 Forward problem for Burger's equation

The balance equation for momentum in fluids with constitutive equation in homogeneous isotropic materials become (the Cauchy stress tensor) Equation (82) (the Cauchy stress tensor) with no external force becomes the Navier—Stokes equations in the form of

(211) $$\rho \left[\frac{\partial v}{\partial t}+\left(v\cdot \nabla \right)v\right]=\mu \;\Delta v+\left[\zeta +\frac{1}{3}\mu \;\right]\nabla \left(\mathrm{div}\;v\right)-\nabla p\text{,}$$

where v(r,t) is the velocity, ρ(r,t) is the density, p(r,t) is the pressure, and *μ* = *μ* (**r**,*t*) and *ζ* = *ζ* (**r**,*t*) are the dynamic and second viscosity coefficients (see Equation (80)). In this case, in 1D, assuming for incompressible fluid flows (constant density, ρ(x,t)=ρ), the Navier—Stokes equations (Equation (94)) become

(212) $$\frac{\partial u}{\partial t}+u\;\frac{\partial u}{\partial x}=\nu \frac{\partial^{2}u}{\partial x^{2}}-\frac{1}{\rho }\;\frac{\partial p}{\partial x}\text{,}$$

where u(x,t) the velocity in the *x* direction (*u* = v_x_), *p* (x,t) is the pressure, and ν is the kinematic viscosity at *x* at time *t*. Assuming constant pressure, we obtain Burger's equation in 1D,

(213) $$\frac{\partial u}{\partial t}+u\;\frac{\partial u}{\partial x}=\nu \frac{\partial^{2}u}{\partial x^{2}}\;\text{.}$$

As an example, we consider the 1D Burger's equation in the domain of x∈[−1,1];t∈[0,1] with initial conditions u(−1,t)=u(1,t)=0 and Dirichlet boundary conditions u(x,0)=−sin(πx).

For the forward problem, we need a reference solution to Burger's equation under the given conditions, otherwise computed. DeepXDE provides such data at this URL:

https://github.com/lululxvi/deepxde/blob/master/examples/dataset/Burgers.npz.

Each term in the PDE is represented in DeepXDE with an appropriate method for invoking auto-differentiation. We instantiated a fully connected network with an input layer consisting of two nodes, three hidden layers with 20 nodes each, and an output layer with a single node. We use “tanh” for the activation function, and “Glorot normal” to randomly initialize the nodes. Once instantiated, the data object is passed to the Model class, along with the instantiated network object.

Next, we train the network. We train the network in two phases. We do this because of the relatively small diffusion coefficient. As this coefficient becomes very small, it becomes increasingly difficult to estimate the solution. Accordingly, for better performance, we use an initial optimization that quickly provides a “rough-cut” solution quickly: *adam* (adaptive moment estimation). Then we continue training with an optimization that provides a more “finely-tuned” solution: *L-GBFS* (limited-memory Broyden–Fletcher–Goldfarb–Shanno).

Next, we define the number of training iterations to consider for each phase. It is possible to get stuck in a local optimum and see no further improvement. In such cases, there is no point in continuing; we want to stop. DeepXDE provides a callback mechanism to do this. The criteria for stopping are chosen by the user as necessary. After completing the first phase of training we proceed to the second phase. As before, we set callbacks to manage the process, then proceed with training.

**Fig. 1:**
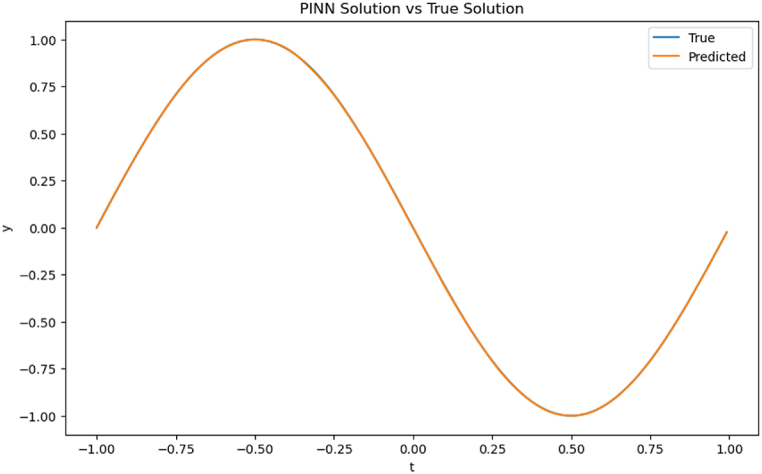
Berger's equation: True vs predicted solution (fixed position); no discernible difference.

**Fig. 2:**
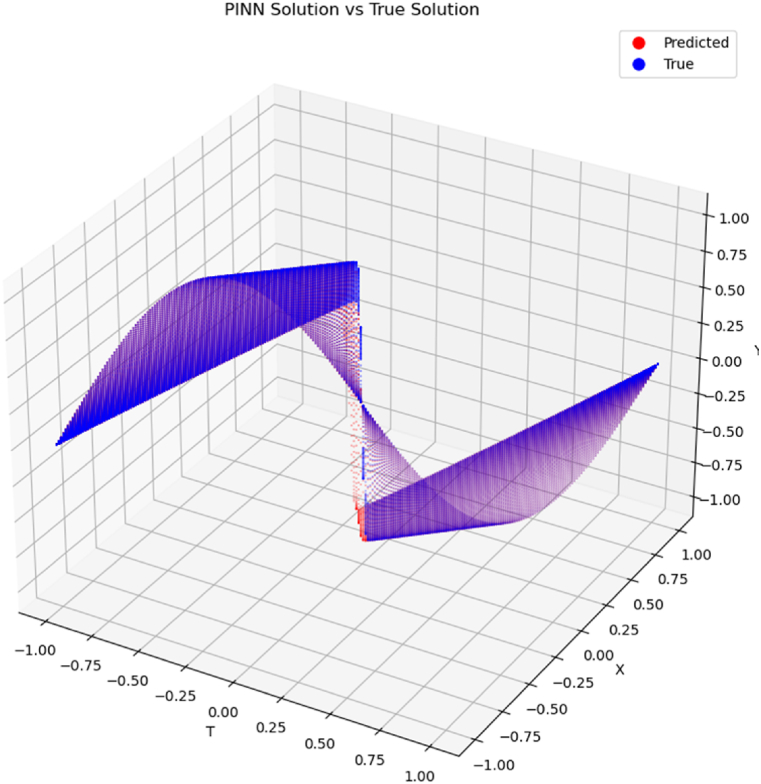
Berger's equation: True vs predicted solution.

Fig. 1 compares the true and predicted solution for a given slice of the position in space. Fig. 2 shows both true and predicted solution for the full domain.

#### 11.1.2 Inverse problem for Burger's equation

Having solved the forward problem for Burger's equation, we can consider the inverse problem using essentially the same element. All we need to do is make the modifications to identify any unknown elements. For simplicity, let us assume that we do not know the diffusion coefficient.

Setting up the inverse problem requires a source of data, presumably generated from Burger's system. These data are used to train the model, selecting the value for the diffusion coefficient that gives the best fit. The code is formally very similar to the forward problem. Instead of specifying the value of the diffusion coefficient as in the forward problem, we initialize diffusion coefficient that we seek to discover. Next, we load data to be used for training. and define *collocation points* for the training, i.e., points within the domain of the problem where the neural network is trained to satisfy the PDE. As before, we construct and initialize the network, but for the data object, identifying the anchors, i.e., the colocation points, and specify the number of points at which to evaluate the residuals. We then compile the model, identifying to the model any unknown variables.

In this case we estimate the kinematic viscosity to be 0.0048, while the true value is ν = 0.0032. We omit the visualizations because the it closely resembles the forward case, i.e., we successfully estimated the unknown parameter with this network design. and ν is the kinematic viscosity

Note that these are *only the changes* that need to be made. Of course, one may wish to introduce callbacks to monitor training progress and/or generate related graphs for visualization.

### 11.2 Hagen—Poiseuille flow

The Hagen—Poiseuille flow (or Poiseuille flow for short) describes the stationary incompressible laminar flow of a viscous fluid through a long, straight tube or pipe with constant cross section generated by a constant pressure gradient along the flow. No slip conditions are assumed for the velocity at the boundary. As our example for PINN, we use the Poiseuille flow in a pipe with a rectangular cross section.

The balance equation for momentum in incompressible fluids with no external force (the Navier—Stokes equations, Equation (91)) becomes

(214) $$\rho \left[\frac{\partial v}{\partial t}+\left(v\cdot \nabla \right)v\right]=\mu \;\Delta v-\nabla p\text{,}$$

Where, for the flux, we used the constitutive equation expressed by the Cauchy stress tensor, Equation (82). Assuming a stationary laminar flow, we obtain

(215) ρ[(v·∇)v]=μΔv−∇p,

where v(r,t) is the velocity, ρ(r,t) is the density, p(r,t) is the pressure, and *μ* = *μ* (**r**,*t*) and *ζ* = *ζ* (**r**,*t*) are the dynamic and second viscosity coefficients (see Equation (80)). Assuming we have a laminar flow with a constant velocity along the *z* direction, we obtain the following elliptic PDE:

(216) $$\mu \left[\frac{\partial^{2}u}{\partial x^{2}}+\frac{\partial^{2}u}{\partial y^{2}}\right]-\frac{\partial p}{\partial z}=0\text{,}$$

where u(x,y) the velocity in the *z* direction (*u* = v_z_), *p* (z) is the pressure with a constant gradient. In our example, we use

no slip conditions at the walls with thickness of 1: u(+/−0.5,y)=u(x,(+/−0.5)=0.

#### 11.2.1 Forward problem for Poiseuille flow

We begin by specifying the system, domain, and constraints. The specification and training of the neural network is essentially the same as we used for Burger's equation. The main difference is that the Poiseuille flow is steady state, i.e. no time dependence. This results in a simpler construction and initialization of the network.

**Fig. 3:**
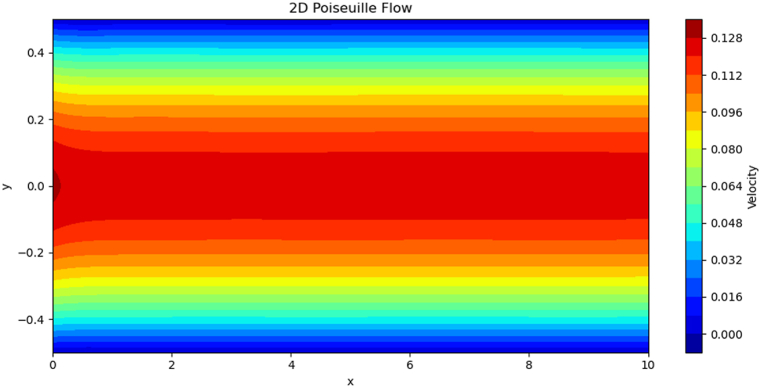
Poiseuille equation: Forward solution.

Our result is shown in Fig. 3. The velocity field displays the expected behavior of laminar flow with zero flow velocity at the pipe boundary and maximum flow velocity in the center of the pipe.

#### 11.2.2 Inverse problem for Poiseuille flow

Next, we consider the inverse problem for the Poiseuille flow. We identify the unknown parameters and represent the PDE using these parameters; there is only one: the unknown pressure gradient. We generate data from a known Poiseuille process, i.e., the forward model we previously trained and the PDE defined with an unknown parameter, the pressure gradient. We construct and initialize the neural network using these data. Finally, then train the model that identifies the unknown variable to be estimated.

**Fig. 4:**
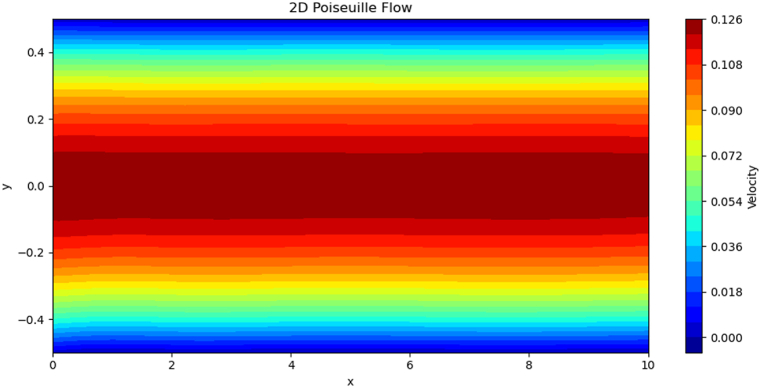
Poiseuille equation: Inverse solution.

Fig. 4 shows our result. The velocity profile is essentially that obtained from the forward solution. The pressure gradient is estimated to be 0.995, while the correct value is 1.0.

### 11.3 Poisson's equation

In this example, we apply our method to the Poisson's equation of electrodynamics. We assume that the magnetic field is negligible, B(r,t)=H(r,t)=0. In this case, the balance equation for electromagnetic energy density in isotropic medium, Equation (148), assuming that the permeability, $\epsilon_{e}$, does not depend on time, becomes

(217) $$\frac{\epsilon_{e}}{2}\;\frac{\partial E^{2}}{\partial t}=-4\pi \;j_{e}\cdot E\text{.}$$

where $j_{e}$ is the electric current. Carrying out the time derivation and pulling the electric field, E(r,t), we obtain

(218) $$E\cdot \;\frac{\partial \epsilon_{e}E}{\partial t}=-4\pi \;j_{e}\cdot E\text{.}$$

Dividing both sides by E(r,t), assuming that it is nowhere zero, using the constitutive relation of electrodynamics, $D\left(r,t\right)=\epsilon_{e}E\left(r,t\right)$, and taking the divergence of both sides, we are led to

(219) $$\frac{\partial }{\partial t}\mathrm{div}\;D=-4\pi \;\mathrm{div}\;j_{e}\text{.}$$

where we switched the order of time derivative and div operator assuming that the function is necessarily smooth. Using the continuity equation (Equation (136)) and integrate it, we obtain

(220) $$\mathrm{div}\;D=-4\pi \;\rho_{e}\text{,}$$

where $\rho_{e}\left(r,t\right)$ is the electric charge density.

Starting from the full balance equation for electrodynamics energy, Equation (147), we obtain a relation for the curl of the electric field. Equation (147) leads to Equation (152), and since B(r,t)=H(r,t)=0, Equation (152) becomes

(221) ∇×E=0.

Any vector field that has curl identically zero, can be written as a gradient of a scalar field, in this case it is called the electric potential, U,

(222) E=−∇U.

Using this in Equation (220), we get the Poisson equation for the electric field:

(223) $$\Delta U=-\frac{\rho_{e}}{\epsilon_{e}}\;\text{.}$$

We can solve this problem using a PINN following the same procedure as before. The essential elements are to specify the differential system to solve, the domain, and constraints. These are then communicated to the PINN via the loss function. The PINN needs to be parameterized to find the solution. To do so accurately and efficiently may require tuning the PINN design, such as the NN architecture, number and size of hidden layers, learning rate, and optimization method, .e.g., “adam”.

## 12 Summary

We have demonstrated that the basic principle of balance enables the derivation of the fundamental equations of various branches of classical physics utilized in analyzing complex systems. Furthermore, we have shown that this principle is essentially a form of bookkeeping process. The principle of balance is universal, as it relies on accurately tracking the sources and fluxes of the relevant extensive quantities generated by the inhomogeneities of their associated intensive quantities.

In general, every continuous system can be described by the conservation equations of three extensive quantities: total mass, momentum, and energy. The local conservation of total mass is encapsulated by the continuity equation, where the relevant extensive quantity is the mass density, and its associated intensive quantity is the chemical potential (Table 2). Assuming the absence of mass sources and inhomogeneities in the associated intensive quantity (i.e., the chemical potential), we derived the mass continuity equation (Section 5.1). The conservation of momentum is governed by the momentum equation, where the density of the relevant extensive quantity is the momentum flux density, and its associated intensive quantity is the velocity, as in fluid mechanics (Section 5), or the displacement vector, as in the case of elasticity (Section 6). In Table 6, we present the components of the momentum flux density tensor for fluid dynamics, elasticity, and electrodynamics, assuming that the fluxes of extensive quantities are proportional to the first derivatives of the relevant inhomogeneous intensive quantities. This assumption is generally valid for most practical applications.

**Table 6:** Fluid dynamics, elasticity, and electrodynamics at a glance. The table contains the components of the momentum flux density tensor, **J** = **J**_cd_ + **J**_cv_, where **J**_cd_ = −**σ** **=** −**τ** + *p*_*eff*_**I**_**3**_, where **σ** is the Cauchy stress tensor.

	Deviatoric tensor (*τ*)	Effective pressure (*p*_*eff*_)	Convective flux density tensor (J_cv_)
Fluid mechanicsa)	2*μ*[**ε**(**v**) − (**1***/***3**)(div **v**)**I**_**3**_]	*p* − *ζ*b) div **v**	*ρ***v** ⊗ **v**
Linear elasticityc)	2*μ*[**ε**(**u**) − (**1***/***3**)(div **u**)**I**_**3**_]	−[*λ* + (1*/*3)*μ*]d) div **u**	0
Electrodynamics	0	[1*/*(8*π*)] [**E** · **D** + **H** · **B**]	[−1*/*(4*π*)] **E** ⊗ **D** + (−1*/*(4*π*)) **H** ⊗ **B**(e)

a **v** is the velocity of the fluid, **ε**(**v**) is the rate of strain tensor (Equation (78)).

b *ζ* = *λ* + (2*/*3)*μ* is the second viscosity coefficient, where *λ* and *μ* are the first and second Lamé parameters, also called the bulk and dynamic viscosities in hydrodynamics (Section 5.2).

c **u** is the displacement vector of elasticity, **ε**(**u**) the elastic strain tensor (Equation (105)).

d *λ* and *μ* are the first and second Lamé parameters (Equation (108)); note that in fluid dynamics *λ* + (1*/*3)*μ* = *ζ*, but *ζ* is not introduced in elasticity.

e Note that convective flux density tensor (**J**_cv_) is not defined in electrodynamics, this expression is based on an analogy (Section 8.2).

Energy conservation is governed by the energy balance equation. In continuum mechanics, the sources of energy are the work done on the system by external body forces and the heat generated within the system. For fluid dynamics and elasticity, the conductive energy flux is associated with the Cauchy stress tensor, and the heat transport is described by Equation (72). The energy fluxes are derived by multiplying the Cauchy stress tensor with the velocity, as detailed in Section 4.3. In the context of electrodynamics, the energy equation is derived using the Poynting vector (Equation (145)), which represents the electromagnetic flux density, as discussed in Section 8.3.

Using the general principle of balance, we derived the basic equations of mechanical and electrical systems: the continuity equation (Section 4.1), the Navier–Stokes equations (Section 5.2), the Navier's equations of elasticity (Section 6.2), Maxwell's equations (Section 8.3), the Euler equations (Section 9.3), and Bernoulli's Equation (Section 9.4).

Simple examples for balance equations for systems with many applications involving only one pair of extensive and intensive quantities are provided in the derivation of Fick's law and Fourier's equation (Sections 9.1, 9.2). As examples of more complicated applications of continuous systems, those involving multiple processes (e.g., mechanical, thermal) described by multiple extensive and intensive variables, we derived the basic equations of fluid mechanics and elasticity (Sections 5, 6).

In Section 10.1, we proposed an algorithm based on the balance equations, which can be integrated in PIML methods to solve complex scientific and engineering problems. This algorithm can be adopted to describe various physical processes by identifying the relevant intensive and extensive variables and their fluxes for the given system. We illustrated our proposed method in Section 10.2 by applying it to analyze a system described by Eulerian hydrodynamics.

In Section 11 we provided worked examples of how the balance equation method can be implemented in PIML. We start from the balance equations and implement a PIML based on DeepXDE. We provided examples for two basic forms of PDEs, the hyperbolic (Burgers equation; Section 11.1) and the elliptic (Hagen-Poiseuille and Poisson's equations; Sections 11.2 and 11.3). Upon written request (e.g., email), we will provide the Deepxde-based codes used for these examples.

We note some limitations to the use of balance equations for PIML. Most importantly, the balance equation method can only be applied to systems with conservation laws, i.e., conservative systems. If a system can be described by fundamental physical principles, conservation laws can be established. If empirical effective terms are required to describe a system, e.g., if it is not feasible (or possible) to return to fundamental principles, then the balance equation method is not applicable. applicable.

In this paper we have presented applications of the differential form of the balance equations. The applicability of this form requires that the system is differentiable, implicitly assuming that the system is local, i.e., fluxes depend only on their immediate neighborhood. We have focused on 2nd order systems. Higher order systems may arise from more complicated source terms. The balance principle can also be applied to non-differentiable systems by using the integral form of the balance equations. The study of integral systems using the PINN technique has recently been considered [63] and is currently an active area of research.

Recent work proposes novel approaches to the inverse problem using reinforcement learning to discover the form of the PDE during the training process using a symbolic representation of the possible terms [64]. In the case of a problem that can be traced back to fundamental physical principles, the balance equation approach constrains the form of the possible terms in the PDE describing the system. In future work, a combination of the balance equation method and reinforcement learning using a symbolic representation of the terms in the PDEs could potentially make the inverse problem more tractable and the solution more efficient.

## 13 Conclusion

We have demonstrated that balance equations can be utilized to design physics modules based on a common algorithm that can be incorporated into physics-informed machine learning (PIML) methods to solve complex scientific and engineering problems. We have also provided worked examples of how the balance equation method can be implemented in PIML. Therefore, complex systems involving various physical processes can be modeled using the same algorithm based on the balance equations. Constraining machine learning methods using balance equations ensures that the results are consistent with the laws of physics.

This paper gathers in one place, with a unified framework, all of classical physics. The paper is exhaustive in terms of balance equations and comprehensive in terms of constitutive equations, though not exhaustive, as new contexts may arise. The constitutive relations are the essential consideration to add semantics to the balance equations. The issues of system domain and constraints are necessarily problem specific and beyond the scope of this paper. The relevance for users of PINN and now PCNN technology is that one has a comprehensive source for specifying physics relevant to machine learning models.

Moving forward, we aim to enhance the proposed balance equation framework by developing a unified algorithm to enforce different types of initial and boundary constraints. We plan to demonstrate how the proposed balance equation can guide the integration of physical laws and constraints into machine learning models to analyze complex systems. We will validate the generalizability of the framework through in-depth case studies across multiple physical and engineering domains.

## CRediT authorship contribution statement

**Sandor M. Molnar:** Writing – review & editing, Writing – original draft, Methodology, Investigation, Formal analysis, Conceptualization. **Joseph Godfrey:** Writing – review & editing, Writing – original draft, Investigation, Conceptualization. **Binyang Song:** Writing – review & editing, Writing – original draft, Investigation, Formal analysis, Conceptualization.

## Declaration of competing interest

The authors declare that they have no known competing financial interests or personal relationships that could have appeared to influence the work reported in this paper.

## APPENDIX

### A. Mathematical notations

In this section, as in the main sections, the indices are either 1, 2, or 3 and the sums over them always run from 1 to 3, e.g., as *i* in $\sum_{i=1}^{3}\text{.}$ We use either (v_*x*_,v_*y*_,v_*z*_) or (v_1_,v_2_,v_3_) for the *x*, *y*, and *z* components of a vector, **v**.

We define the multiplication of a 2nd-rank tensor, **T**, with a vector, **v**,

(A1) $${\left(Tv\right)}_{i}=\sum_{j}T_{ij}\;v_{j}\text{.}$$

Wherever it is convenient and it does not lead to confusion, we use the “nabla” operator (∇) for partial derivatives,

(A2) $$\nabla =\left(\partial_{x},\partial_{y},\partial_{z}\right)=\left(\partial_{1},\partial_{2},\partial_{3}\right)\text{.}$$

or ∇_*i*_ = *∂/∂x*_*i*_ for its *i*th component.

For the gradient operator acting on a scalar field, *s*(**r**,*t*), we use one of the following expressions:

(A3) $$\text{grad}\;s\left(r,t\right)=\nabla s\left(r,t\right)=\left(\partial_{x},\partial_{y},\partial_{z}\right)\;s\left(r,t\right)=\left(\partial_{1},\partial_{2},\partial_{3}\right)\;s\left(r,t\right)\text{.}$$

For the divergence of a vector field, v(**r**,*t*), we use one of the followings:

(A4) $$\mathrm{div}\;v\left(r,t\right)=\nabla \cdot v=\partial_{x}v_{x}+\partial_{y}v_{y}+\partial_{z}v_{z}=\sum_{i}\partial_{i}v_{i}\text{.}$$

For the Laplace differential operator, Δ = ∇ · ∇, acting on a scalar field we use one of the following expressions,

(A5) $$\Delta s\left(r,t\right)=\partial_{x}^{2}s+\partial_{y}^{2}s+\partial_{z}^{2}s=\left[\sum_{i}\partial_{i}^{2}\right]s\left(r,t\right)\text{,}$$

acting on a vector field, the vector Laplacian, we use

(A6) $$\Delta v\left(r,t\right)=\left[\sum_{i}\partial_{i}^{2}v_{1},\sum_{i}\partial_{i}^{2}v_{2},\sum_{i}\partial_{i}^{2}v_{3}\right]\text{.}$$

The gradient of a vector field, **v**(**r**,*t*), is a 2nd rank tensor field, defined as

(A7) $${\left(\text{Grad}\;v\right)}_{ij}=\frac{\partial v_{i}}{\partial x_{j}}\text{.}$$

We use double-dot, *S* = **A**:**B**, for scalar products of two tensors of the same rank (**A** and **B**). For example, the scalar products of 2 s rank tensors can be written as

(A8) $$S=\sum_{k,l}A_{kl}B_{kl}\text{,}$$

where *k* and l = 1, 2, 3 for 3D tensors.

### B. Classification of partial differential equations

The classification of by 2nd order partial differential equations (PDEs) is based on how their solutions look like in some simple cases. In many cases physical systems can be represented by 2nd order PDEs,

$$\sum_{i,j}^{n}a_{ij}u_{ij}+b_{i}u_{i}+cu=d;\;u_{i}=\partial_{i}u\;;\;u_{ij}=\partial_{ij}u\;;\;u=u\left(q\right)\;;\;q\in \Omega \subseteq R^{n}\text{.}$$

The constants $A=\left[a_{ij}\right]$, $B=\left[u_{i}\right]$, c, and d can be generalized to be known functions, but for our exposition here we assume these are constant.

These differential equations are classified according to the properties of the matrix A. PDE's are classified by considering the eigenvalues of the determinant

$$\left|A-\lambda I\right|=0\;;\;I=\left[\delta_{ij}\right]\;;\;\delta_{ij}=\left\{ \begin{array}{cc} 1 & i=j\\ 0 & i\neq j \end{array}\right.\;\text{.}$$

Let $e_{>}$ and $e_{0}$ denote the number of positive and zero eigenvalue, respectively. Then PDE's are classified as:

$$Hyperbolic\;{\;e}_{0}{=0,e}_{>}=1;\;e_{0}{=0,e}_{>}\leq n-1$$

$$Parabolic\;{\;e}_{0}>0,i.e.,\left|A\right|=0$$

$$Elliptic\;e_{0}{=0,e}_{>}=n;\;e_{0}{=0,e}_{>}=0$$

where n is the dimensionality of the domain.
